# Artificial human Met agonists based on macrocycle scaffolds

**DOI:** 10.1038/ncomms7373

**Published:** 2015-03-11

**Authors:** Kenichiro Ito, Katsuya Sakai, Yoshinori Suzuki, Naoya Ozawa, Tomohisa Hatta, Tohru Natsume, Kunio Matsumoto, Hiroaki Suga

**Affiliations:** 1 Department of Chemistry, Graduate School of Science, The University of Tokyo, Tokyo 113-0033, Japan; 2 Division of Tumor Dynamics and Regulation, Cancer Research Institute, Kanazawa University, Kanazawa 920-1192, Japan; 3 National Institute of Advanced Industrial Science and Technology, Biological Information Research Center, Tokyo 135-0064, Japan

## Abstract

Hepatocyte growth factor (HGF) receptor, also known as Met, is a member of the receptor tyrosine kinase family. The Met–HGF interaction regulates various signalling pathways involving downstream kinases, such as Akt and Erk. Met activation is implicated in wound healing of tissues via multiple biological responses triggered by the above-mentioned signalling cascade. Here we report the development of artificial Met-activating dimeric macrocycles. We identify Met-binding monomeric macrocyclic peptides by means of the RaPID (random non-standard peptide integrated discovery) system, and dimerize the respective monomers through rational design. These dimeric macrocycles specifically and strongly activate Met signalling pathways through receptor dimerization and induce various HGF-like cellular responses, such as branching morphogenesis, in human cells. This work suggests our approach for generating dimeric macrocycles as non-protein ligands for cell surface receptors can be useful for developing potential therapeutics with a broad range of potential applications.

## Abstract (web summary)

Activation of the Met receptor by hepatocyte growth factor requires Met receptor dimerization. Here, the authors identify Met-binding peptide macrocycles that, in a dimeric form as a result of chemical crosslinking, induce Met receptor dimerization and activation in cultured human cells.

## Graphical abstract

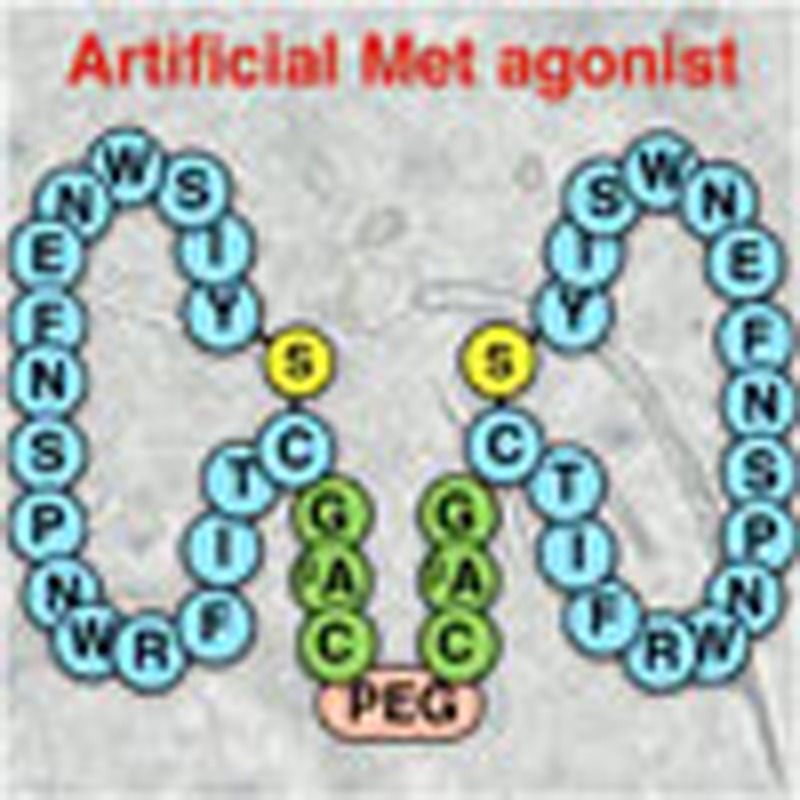

Receptor tyrosine kinases (RTKs) belong to a family of single transmembrane receptors and play upstream regulatory roles in various cellular signalling pathways^1^. RTKs are generally stimulated by growth factors or hormones, and these specific RTK–ligand interactions trigger important cellular activities, such as migration, proliferation and morphogenesis. Hepatocyte growth factor (HGF) receptor, also known as Met or c-Met, is a class IV RTK that interacts with its specific ligand, HGF, through the Met ectodomain (extracellular domain) to form Met–HGF dimers^2^,^3^,^4^. This event brings the respective intracellular tyrosine kinase domains into close proximity^5^,^6^, promoting *trans*-phosphorylation (autophosphorylation) of tyrosine residues. These phosphorylated forms then activate various signal transducers, such as Grb2, Src, Gab1, Akt and Erk^3^,^4^,^7^,^8^ (Fig. 1a).

The therapeutic implications of the Met signalling cascade are twofold. By affecting the above-mentioned downstream kinase-mediated pathways, abnormal Met activation promotes oncogenesis in diverse tissues. Thus, interference with the Met–HGF interaction is considered a potential preventative measure against oncogenesis and malignant transformation^9^,^10^. On the other hand, Met plays a critical role in embryonic development and wound healing. Therefore, Met-activating molecules are potentially useful agents in regenerative medicine^11^. In fact, human recombinant HGF (hHGF) and hHGF gene therapy (Collategene, a hHGF DNA plasmid) have been examined as a treatment option in several intractable diseases, such as amyotrophic lateral sclerosis, hepatic cirrhosis and peripheral arterial disease^11^,^12^. Although therapeutic to some extent, these approaches may be limited by a risk of unpredictable immunological response in patients. Thus, alternative drug candidates (ideally smaller molecules) that can activate the Met signalling cascade are evidently required.

Here we report dimeric macrocyclic peptides that induce Met dimerization and thereby activate downstream Met signalling cascades. Anti-Met macrocycles, discovered by the use of the RaPID (random non-standard peptide integrated discovery) system^13^ (Fig. 1b), exhibit strong affinity to Met with dissociation constants (*K*_D_) ranging from a few to 20 nM. Although these macrocycles cannot activate Met signalling *per se*, they become effective agonists after dimerization by appropriate cross-linkers and specifically activate Met-related downstream signalling pathways. Cellular responses such as proliferation, migration and branching morphogenesis are induced at dimeric macrocycle concentrations of <10 nM and reach maximal effect at about 100 nM. In summary, through the engineering of artificially selected macrocycles, we have developed agonists of a receptor-mediated signalling cascade, opening a unique opportunity to develop ‘non-protein’ regenerative medicines.

## Results

### RaPID selection of anti-Met macrocycles and *in vitro* characterizations

The RaPID system consists of the following techniques: (1) the flexizyme integrated translation (FIT) system^14^ that facilitates genetic code reprogramming of non-proteinogenic amino acids and (2) messenger RNA display^15^,^16^ that fuses the expressed peptide (phenotype) with its cognate mRNA (genotype), enabling amplification of activity-enriched phenotypes (Supplementary Fig. 1). In this study, we reassigned the initiator fMet to ClAc^L^Y (*N*-chloroacetyl-L-Tyr) or ClAc^D^Y (*N*-chloroacetyl-D-Tyr) in the FIT system and used an mRNA library with a complexity of 10^14^ that was designed to have random sequences designated by (NNK)_*n*_, (*n*=4–15) flanked by the initiator AUG (ClAc^L/D^Y) and UGC (Cys) codons, followed by a short linker peptide. On expression of the respective mRNA library using the ClAc^L^Y or ClAc^D^Y initiator, linear peptides exceeding 10^12^ unique members self-cyclized by reaction of the amino-terminal ClAc group and the sulfhydryl group of a downstream Cys residue to yield sulfide-macrocycles. Although the peptide library was constructed from a single mRNA library, the cyclic scaffold ensured that the D-library (whose members contain a single ^D^Y at the N-terminus and other elongating amino acids are L-form) and the L-library (comprising all L-amino acids with ^L^Y at the N-terminus) covered different three-dimensional spaces. Applying these libraries, we then selected ligands against the ectodomain of human Met immobilized on Protein G magnetic beads via an Fc-tag.

The recovery of peptide–mRNA fusion molecules was significantly increased following four and five rounds of selection using the L- and D-libraries, respectively (as compared with the background recovery resulting from nonspecific binding to the magnetic beads alone; Supplementary Fig. 2), suggesting that the population of Met-binding macrocycles was selectively enriched. Sequence analysis of the respective enriched libraries revealed numerous unique sequences (Supplementary Table 1) in which we found some conserved motifs, such as YWYY or PYFXV(I/V) in the L-library and VWXLD in the D-library. We selected the most abundant clones from the respective libraries, aML5, aMD4 and aMD5 (named after the anti-Met L- or D-number of the cloned peptide), for further characterization (Supplementary Table 2).

The respective macrocycles were characterized *in vitro* by surface plasmon resonance (SPR) and all exhibited strong affinity to the Met ectodomain (*K*_D_=2–19 nM; Supplementary Fig. 3a). The *K*_D_ values of their linear counterparts (aML5-Lin, aMD4-Lin and aMD5-Lin) were approximately two orders of magnitude higher than that of the parental peptides (Supplementary Table 2 and Supplementary Fig. 3b), indicating that the macrocyclic scaffold is critical for binding affinity. Moreover, scrambling the sequence of aMD4 (aMsD4) resulted in complete loss of its binding activity.

We also examined whether the macrocycles could cross-bind to murine and canine Met ectodomains, in addition to the human Met ectodomain used for selection. Although murine and canine Met are known to cross-bind to hHGF, we did not observe hHGF binding when the murine or canine ectodomains were directly immobilized via amine coupling on the SPR sensor chip. As this failure might be caused by denaturation of the ectodomains during the immobilization process, we prepared biotinylated hHGF or macrocycles and immobilized the respective ligands on a streptavidin sensor chip. In these experiments, we were able to detect binding of the immobilized hHGF to all three of the ectodomains (human, canine and murine) tested with only subtle differences in response. However, although the macrocycles immobilized on the SPR sensor chip showed strong binding profiles to the human ectodomain, binding was detected to neither the murine nor the canine ectodomains (Supplementary Table 3 and Supplementary Fig. 4). We also found that the macrocycles did not compete with hHGF for binding to Met (Supplementary Fig. 5a). The above evidence, along with no observed sequence similarity between the macrocycles and hHGF, suggests that the Met-binding site(s) of the macrocycles probably differs from that of hHGF.

### Binding of macrocycles to Met on live cells

We next evaluated whether the macrocycles could bind to Met on live cells. For this purpose, we adopted two approaches: (1) a proteomic approach using FLAG-tagged macrocycles and (2) live-cell imaging using fluorescently labelled macrocycles.

To conduct pull-down experiments for proteomic analysis of interacting proteins, we modified the macrocycles to include a FLAG-tag. As the respective macrocycles were selected as peptide–mRNA fusions via a carboxy-terminal (GS)_3_-linker, each peptide’s C-terminus appeared likely to tolerate not only FLAG modification but also binding to an anti-FLAG monoclonal antibody (mAb) without compromising the structure of the active macrocyclic region. Therefore, we chemically synthesized the relevant macrocycles with C-terminal (β-alanine)_2_-FLAG tags (βA_2_-FLAG), with the resulting peptides referred to as aML5-FLAG, aMD4-FLAG and aMD5-FLAG (Supplementary Table 2 and Supplementary Fig. 3c). We confirmed that all of these macrocycles with βA_2_-FLAG tags retained sufficient binding activity to the Met ectodomain, with only modest increases in *K*_D_ values. HepG2 cells incubated with these FLAG-tagged ‘bait’ macrocycles were washed and lysed, and ‘prey’ proteins bound to the bait were immunoprecipitated with an anti-FLAG mAb immobilized on agarose beads. Following endopeptidase digestion of the isolated proteins, the peptide fragments were analysed by direct nano-flow LC–MS/MS (liquid chromatography–tandem mass spectrometry)^17^. When the bait ligand was aML5-FLAG, only a single Met fragment was isolated (Supplementary Table 4), indicating that it selectively bound to Met. Similarly, the aMD4-FLAG bait was able to capture several Met fragments along with two fragments of PDIA3 (a protein disulfide isomerase family A, member 3). Unfortunately, the aMD5-FLAG bait captured only a fragment of NDUFA5 (NADH dehydrogenase 1α subcomplex subunit 5) instead of Met fragments. The reason for this unexpected result is yet to be fully elucidated; however, it is possible that the mAb binding to the C-terminal FLAG tag of this particular macrocycle hinders the macrocyclic portion from interacting with Met. This idea is consistent with our data of dimerization of aMD5 that requires a long linker, to induce agonistic activity (*vide infra*). Thus, it is likely to be that poor Met binding of aMD5-FLAG-mAb resulted in the unexpected capture of a background protein via nonspecific interactions. Nevertheless, the data generated by the proteomic analysis provide evidence for the selective Met binding of aML5-FLAG and aMD4-FLAG on HepG2 cells.

**Figure 2:**
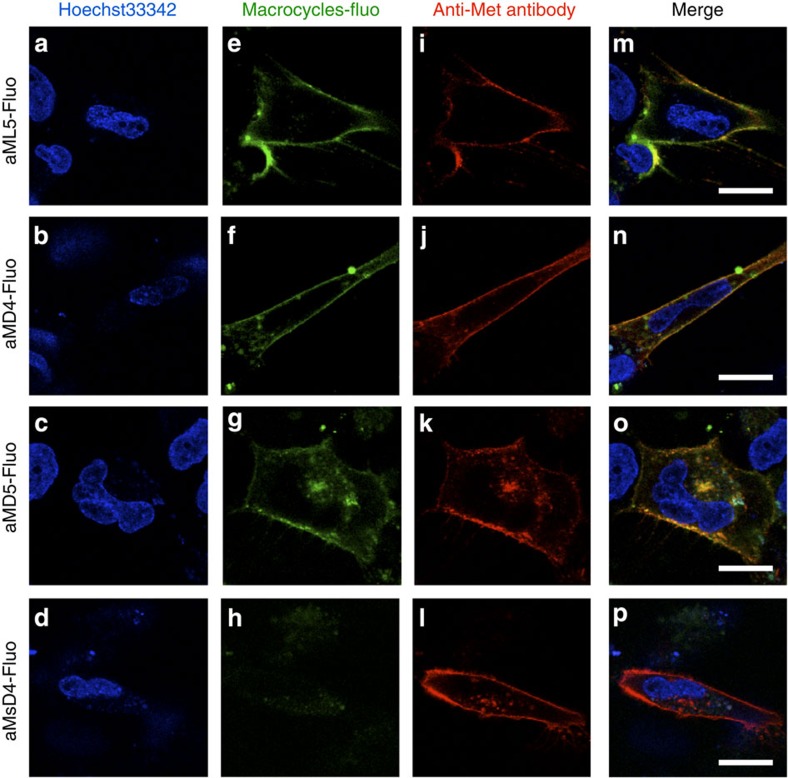
Fluorescein-conjugated macrocycles localized on the cellular membranes of live cells. EHMES-1 cells transiently overexpressing Met were treated with (**a**–**d**) Hoechst33342 and macrocycle-Fluo as follows: (**e**) aML5-Fluo, (**f**) aMD4-Fluo, (**g**) aMD5-Fluo, or (**h**) aMsD4-Fluo. (**i**–**l**) Cell surface Met stained with an anti-Met rat mAb and an anti-rat mAb-Alexa-594 conjugate. (**m**–**p**) Superimposed images of macrocycle-Fluo (green) and anti-Met mAb (red) fluorescent signals. Scale bars, 20 μm.

Next, we examined whether the anti-Met macrocycles selectively bound to the native Met ectodomain on live cells. We exploited the secured C-terminal modification site of each peptide, as discussed above, and prepared fluorescently labelled macrocycles aML5-Fluo, aMD4-Fluo and aMD5-Fluo via the ε-amino group of a lysine residue in a tag peptide at the C-terminus (Supplementary Table 2 and Supplementary Fig. 3d). As expected, the *K*_D_ values of these peptides were nearly identical to those of their parent peptides in Met ectodomain SPR measurements, whereas a control peptide derived from the scrambled aMD4 sequence (aMsD4-Fluo) showed no detectable binding. The respective macrocycles and a Met-specific mAb were co-incubated with EHMES-1 cells transiently overexpressing Met. Subsequently, the cells were treated with an anti-rat mAb-Alexa-594 conjugate (Fig. 2). The fluorescent signal of the membrane stained by macrocycle-Fluo (Fig. 2e–h) coincided with that of the membrane stained by the mAb-Alexa-594 in each case (Fig. 2i–p). By contrast, cells not stained by the mAb-Alexa-594 (that is, cells not overexpressing Met) were also not stained by the macrocycle-Fluo molecules (Supplementary Fig. 6) and the inactive aMsD4-Fluo did not stain cells that were stained by the mAb-Alexa-594 (Fig. 2h,l,p). Collectively, these data, along with the proteomics data, support the view that the macrocycles tested selectively bound to Met expressed on live cells.

### Dimeric macrocycles act as Met agonists

**Figure 3:**
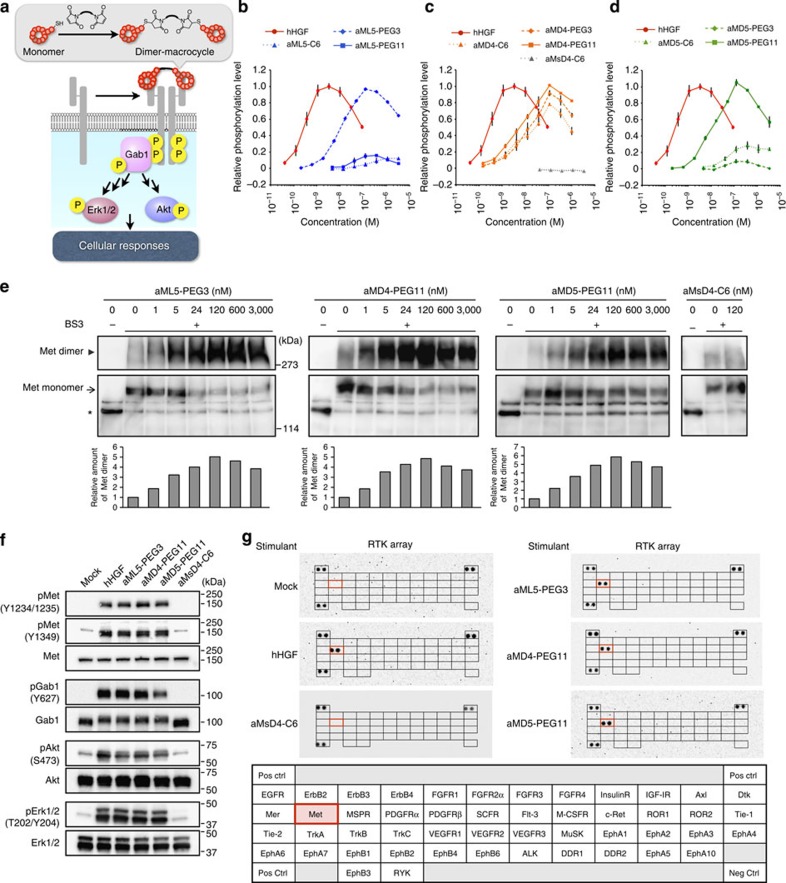
Molecular-level activation of Met signalling pathway by dimeric macrocycles. (**a**) Met activation by the dimeric macrocycles initiates pathways analogous to hHGF-activated pathways. Each dimeric macrocycle was chemically synthesized by conjugating the C-terminal Cys residues using a bis-maleimide cross-linker (Supplementary Table 2) and forms a 1:2 stoichiometric complex with Met. (**b**–**d**) Met phosphorylation level as a function of hHGF concentration (red circles) as a positive control; (**b**) aML5-C6 (blue triangles), aML5-PEG3 (blue rhombuses) and aML5-PEG11 (blue squares); (**c**) aMD4-C6 (orange triangles), aMD4-PEG3 (orange rhombuses), aMD4-PEG11 (orange squares) and aMsD4-C6 (grey triangles) as a negative control; and (**d**) aMD5-C6 (green triangles), aMD5-PEG3 (green rhombuses) and aMD5-PEG11 (green squares). The phosphorylation level of Met in each experiment was quantified by a cell-based phospho-Met ELISA 10 min after cell stimulation. s.d.was calculated from the results of triplicate experiments. (**e**) Analysis of Met dimerization by cross-linking of Met dimer on live cells. EHMES-1 cells were treated with dimeric macrocycles for 60 min at 4 °C. After washing, the cell surface proteins were cross-linked by BS3 cross-linker for 60 min at 4 °C. Cell lysates were subjected to immunoprecipitation and western blotting using an anti-Met antibody. Arrowheads and arrows indicate Met dimer and monomer corresponding to cross-linked αβ subunits, respectively. Asterisks indicate Met monomer corresponding to β-subunit. Each bar in the graph indicates a relative value of the cross-linked Met dimer generated by the band intensity. (**f**) Phosphorylation of Met and downstream signalling proteins. Starved EHMES-1 cells were treated with hHGF (2 nM) or dimeric macrocycles (each at 100 nM) for 10 min and phosphorylation of the respective proteins was analysed by western blotting using their specific antibodies. (**g**) Phosphorylation of various RTKs stimulated by dimeric macrocycles. Lysates of EHMES-1 cells stimulated by 100 nM of dimeric macrocycles or 2 nM of hHGF for 10 min were analysed by a phospho-RTK array of 49 representative human RTKs. Both hHGF and dimeric macrocycles specifically phosphorylated Met (delineated by red boxes).

The intermolecular interaction of hHGF with the Met ectodomain triggers Met dimerization and activates downstream signalling via intracellular *trans*-phosphorylation^4^. Although the anti-Met macrocycles described above might be incapable of inducing dimerization, we reasoned that they might be rendered chemically amenable to induction of dimerization via an appropriate cross-linker (Fig. 3a), a concept known as chemically induced dimerization^18^. To apply this methodology to the anti-Met macrocycles, we designed precursor macrocycles bearing a short peptide motif containing a Cys residue at the C terminus and modified the sulfhydryl group with three bis-maleimide cross-linkers with different lengths, C6, PEG3 and PEG11 (Supplementary Table 2). The apparent *K*_D_ values of the dimeric macrocycles determined by SPR remained in a similar range to the parental peptides (Supplementary Table 2 and Supplementary Fig. 3e).

To test whether these dimeric macrocycles behave as Met agonists, we first monitored phosphorylation of the Y1234/1235 residues in the activation loop of the Met tyrosine kinase domain. Phosphorylation of these residues is an initial hallmark of Met activation^19^. EHMES-1 cells were stimulated by the respective dimeric macrocycles and the phosphorylation level was quantified by enzyme-linked immunosorbent assay (ELISA) using a rabbit pY1234/1235-specific mAb. Appropriate concentrations of dimeric macrocycles were found to stimulate the phosphorylation of Y1234/1235 (Fig. 3b–d), whereas the parental monomer macrocycles were completely inactive (Supplementary Fig. 5b). Intriguingly, appropriate linkers differed among the dimeric macrocycles: (1) aML5 yielded the highest activity when dimerized by PEG3 (aML5-PEG3) and was nearly inactive when dimerized by the other two linkers (Fig. 3b); (2) the activity of dimerized aMD4 was almost independent of the linker length (Fig. 3c), although aMD4-PEG11 appeared slightly more active; and (3) aMD5 dimerized by the longest PEG11 linker (aMD5-PEG11) showed the highest activity, whereas the others were poor agonists (Fig. 3d). These results suggest that the respective macrocycles probably bind different regions of the Met ectodomain, requiring an adequate length of cross-linker for their effective dimerization of Met. We also confirmed that treatment with aMsD4-C6, which is incapable of binding to Met (Supplementary Table 2 and Supplementary Fig. 3e), showed no sign of Met phosphorylation (Fig. 3c). Most importantly, the dimeric macrocycles achieved the same maximal phosphorylation as hHGF, indicating that they can act as fully functional agonists of Met. Notably, elevated concentrations of the dimeric macrocycles (above 100 nM) decreased the observed level of Met phosphorylation and this ‘bell-shaped’ titration response was also observed with elevated concentrations of hHGF (above 5 nM). As it is known that transactivation of Met can occur via epidermal growth factor (EGF)-dependent EGF receptor (EGFR) activation or through a constitutively activated EGFR pathway^20^, we examined whether inhibition of EGF-induced phosphorylation of EGFR by the dimeric macrocycles caused the ‘bell-shaped’ response mentioned above. Addition of 3,000 nM of the representative dimer macrocycle, aML5-PEG3, did not prevent EGF-induced phosphorylation of EGFR (Supplementary Fig. 7), ruling out a mechanism in which inhibition of EGFR activation indirectly causes the suppression of Met phosphorylation.

To further confirm the observed Y1234/1235 phosphorylation, we performed in-cell imaging of Met phosphorylation, using the most active dimeric macrocycles, and monitoring by immunofluorescence microscopy of EHMES-1 cells (Supplementary Fig. 8). The fluorescent imaging data obtained were consistent with the induction of Met phosphorylation at the Y1234/1235 site, indicating that the dimeric macrocycles actively promoted the initial hallmark phosphorylation.

Dimerization of Met induced by HGF is the critical first step for triggering the *trans*-phosphorylation of intracellular kinase domains. If the above-observed Met phosphorylation induced by dimeric macrocycles resulted from the same mechanism, we should also be able to observe similar formation of Met dimers on cell surface. To confirm this, we applied a chemical cross-linking method^21^ to stabilize the Met dimer on cells and detected the corresponding bands by western blotting. As expected, the addition of the dimeric macrocycles produced a slower migrating band compared with the band of monomeric Met, indicating that the cross-linked Met dimer was formed (Fig. 3e, aML5-PEG3, aMD4-PEG11 and aMD5-PEG11). On the other hand, no Met dimerization occurred by the addition of the non-agonistic aMsD4-C6 (Fig. 3e, aMsD4-C6). Notably, the bell-shaped dose–response profiles seen in this Met dimerization assay with both dimeric macrocycles and hHGF (Supplementary Fig. 9) correlated well with those observed for Met phosphorylation (Fig. 3b–d).

Met activation is known to trigger a downstream signalling cascade involving phosphorylation of signature proteins (Fig. 3a). Therefore, we traced the phosphorylation of the downstream adapter protein Gab1 and the cytoplasmic kinases Akt and Erk1/2 (mitogen-activated protein kinase) by means of western blotting, using appropriate specific antibodies. When EHMES-1 cells were stimulated with hHGF or the dimeric macrocycles, Met was phosphorylated at the Y1234/1235 and Y1349 residues (an important signature for the interaction of Met with Gab1 (ref. 22)). In addition, phosphorylations of Gab1 Y627, Akt S473 and Erk1/2 T202/Y204 were observed (Fig. 3f). In each case, the intensity of phosphorylation observed in response to stimulation with each respective dimeric macrocycle were almost identical to that observed following hHGF stimulation (Supplementary Fig. 10). In contrast, the inactive aMsD4-C6 dimer did not induce detectable phosphorylation events, consistent with the hypothesis that the Met-binding activity of the macrocyclic portions of the dimer are crucial for Met dimerization triggering subsequent phosphorylation of Met as well as the downstream proteins.

As downstream phosphorylations might be triggered by other RTKs^1^, we verified whether the dimeric macrocycles selectively activated Met using a phospho-RTK array kit. Cell lysates stimulated by hHGF or the respective dimeric macrocycles were analysed by using the kit. Among 49 RTKs, the dimeric macrocycles at an optimal concentration (100 nM, see Fig. 3b–d) clearly induced phosphorylation of Met alone (Fig. 3g). Notably, MSPR (macrophage stimulating protein receptor, also called RON), which is the closest family of Met in class VI, was not phosphorylated, demonstrating the remarkable Met specificity of the dimeric macrocycles. Based on these data, together with the earlier studies on the specificity of monomeric macrocycle binding to Met, we conclude that the observed downstream phosphorylation events are triggered by the specific activation of Met by the dimeric macrocycles.

We next further investigated whether the observed bell-shaped profile induced by high concentrations of dimeric macrocycles (3,000 nM) or HGF (30 nM) might be due to off-target inhibition of constitutively activated RTKs, which could potentially transactivate Met phosphorylation. We first monitored the phosphorylation levels of RTKs under the same conditions as those used in the experiments in Fig. 3b–d, in the presence of 10% fetal bovine serum (FBS), which might contain various growth factors and receptor ligands. Under such conditions, several RTKs including EGFR, ErbB3, Axl, PDGFRβ, RYK and Met were phosphorylated to some degree (see Mock in Supplementary Fig. 11), suggesting that these receptors were activated by components of the FBS. Under these conditions, addition of an excess of hHGF or aML5-PEG3 strongly induced Met phosphorylation; however, neither affected the phosphorylation level of the RTKs that were observed to be phosphorylated in FBS-containing media alone (Supplementary Fig. 11). To our surprise, phosphorylation of c-Ret was observed following addition of hHGF or aML5-PEG3 to FBS-containing media, despite this phenomenon not being observed in media lacking FBS (Fig. 3g). We hypothesized that such c-Ret phosphorylation might result from transactivation as a consequence of the Met phosphorylation induced by hHGF or aML5-PEG3 in combination with a certain ligand present in the FBS. This hypothesis was supported by the fact that the addition of hHGF or aML5-PEG3 in FBS-containing media induced c-Ret phosphorylation in a dose-dependent manner (Supplementary Fig. 12a), whereas addition of an inhibitor of Met phosphorylation (SU11274) decreased c-Ret phosphorylation, also in a dose-dependent manner (Supplementary Fig. 12b). These results clearly indicate that the observed induction of c-Ret phosphorylation by hHGF and aML5-PEG3 relies on Met phosphorylation, although we are not certain which factor(s) present in FBS plays a role in this transactivation event.

### Dimeric macrocycles induce Met activated cellular phenotypes

The activation of Met signalling induces unique biological responses such as proliferation^23^,^24^, migration^23^,^25^ and morphogenesis^22^,^26^. An important signature of regenerative drugs is the ability to produce such phenotypic cellular changes. To evaluate the potential utility of the described dimeric macrocycles in the repair of damaged cells and in organ tissue regeneration, we next examined whether these compounds were able to elicit phenotypic changes in cells.

To see whether the dimeric macrocycles could stimulate cellular proliferation via activation of the Met signalling pathway, we first used HCC827 cells as a model system. When HCC827 cells were treated with an EGFR-specific inhibitor (gefitinib), their proliferation was strongly suppressed (Supplementary Fig. 13a). However, cellular proliferation could be restored in a dose-dependent manner by treatment with hHGF (Supplementary Fig. 13a,b). Similarly, treatment of these cells with the Met-activating dimeric macrocycles aML5-PEG3, aMD4-PEG11 or aMD5-PEG11 restored cellular proliferation to a similar degree (Supplementary Fig. 13a,b). In contrast, the Met-inactive aMsD4-C6 did not induce proliferation, suggesting that the restored proliferation of HCC827 cells resulted from Met activation by the dimeric macrocycles.

**Figure 4:**
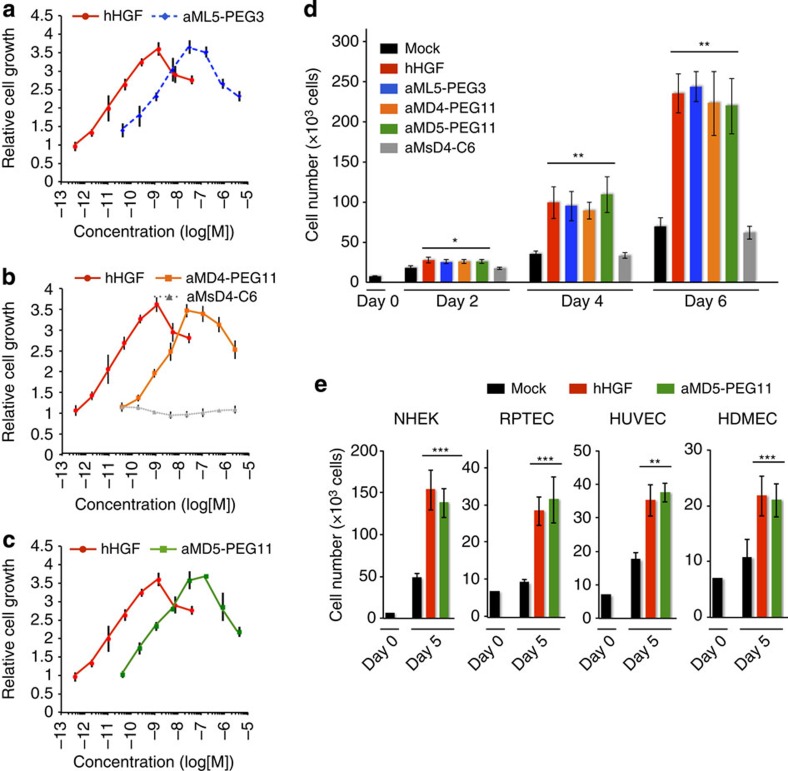
Cellular proliferation promoted by the dimeric macrocycles in normal human cells. (**a–c**) Dose-dependent titration of proliferation induction in normal human epidermal keratinocytes (NHEK) against concentration of hHGF (red) as a positive control, (**a**) aML5-PEG3 (blue), (**b**) aMD4-PEG11 (orange) and aMsD4-C6 (grey) as a negative control, or (**c**) aMD5-PEG11 (green). NHEK were cultured for 6 days with or without hHGF (0.4 pM–30 nM) or dimeric macrocycles (40 pM–3,000 nM). Cells were stained by calcein-AM and quantified by fluorescence intensity. s.d. was calculated from the results of experiments in triplicate. (**d**) Time course of proliferation induction in NHEK. NHEK were cultured with or without 1.3 nM hHGF or 50 nM dimeric macrocycles. After 2, 4 and 6 days, cell numbers were counted by means of an automated cell counter (mean±s.d., *n*=3). *P<0.05, **P<0.01 (unpaired Student’s *t*-test) compared with Mock. (**e**) Proliferation of various normal human cells stimulated by hHGF and aMD5-PEG11. HDMEC, normal human dermal microvascular endothelial cells; HUVEC, normal human umbilical vein endothelial cells; RPTEC, normal human renal proximal tubule epithelial cells. The respective cells were cultured with or without 1.3 nM hHGF or 50 nM aMD5-PEG11. After 5 days, cell numbers were counted by means of an automated cell counter (mean±s.d., *n*≥4). **P<0.01, ***P<0.001 (unpaired Student’s *t*-test) compared with Mock.

Encouraged by the above results, we next evaluated the ability of the dimeric macrocycles to induce the proliferation of normal human cells. We first titrated an extensive range of concentrations of each dimeric macrocycle to determine the optimal concentration to induce proliferation in normal human epidermal keratinocytes (NHEK). All of the dimeric macrocycles tested were able to promote proliferation of NHEK in a dose-dependent manner, with maximal levels of induction comparable to that observed for hHGF (Fig. 4a–c). Again, the binding-inactive aMsD4-C6 showed no effect in these assays (Fig. 4b). The ED_50_ (50% effective dose) of the respective dimeric macrocycles were in the range 1.2–2.7 nM, whereas maximal induction of cell proliferation was seen in the range 50–100 nM. It should be noted that the bell-shaped responses observed in these dose–response studies were similar to those observed in the Met phosphorylation and dimerization studies (Fig. 3b–e). During a 6-day culture period, the dimeric macrocycles at a concentration of 50 nM continuously promoted cell proliferation with comparable efficacy to hHGF at a concentration of 1.3 nM (Fig. 4d). We further attempted to stimulate the proliferation of various normal human cells; NHEK, renal proximal tubule epithelial cells (RPTEC), human umbilical vein endothelial cells (HUVEC) and human dermal microvascular endothelial cells (HDMEC), using a representative dimeric macrocycle, aMD5-PEG11 (Fig. 4e). We observed strong induction of proliferation for all of the above cells in the presence of aMD5-PEG11, with proliferation levels nearly the same as those induced by the control hHGF. Thus, we conclude that the dimeric macrocycles we describe are able to induce the proliferation of normal human cells.

To confirm that the cellular proliferation induced by the dimeric macrocycles involved the promotion of DNA synthesis in response to Met signalling, we monitored DNA synthesis in normal HPAEC (human pulmonary artery endothelial cells). The cells were stimulated by the respective dimeric macrocycles, the negative control aMsD4-C6, or the positive control hHGF. After 20 h of culture with 5-bromo-2′-deoxyuridine (BrdU), quantification of BrdU incorporation into nucleic DNA (Supplementary Fig. 14) indicated that the dimeric macrocycles and hHGF all promoted DNA synthesis, but that aMsD4-C6 did not. This result provided evidence that the dimeric macrocycles promoted DNA synthesis in a similar manner to hHGF^27^ in response to Met-signalling activation.

**Figure 5:**
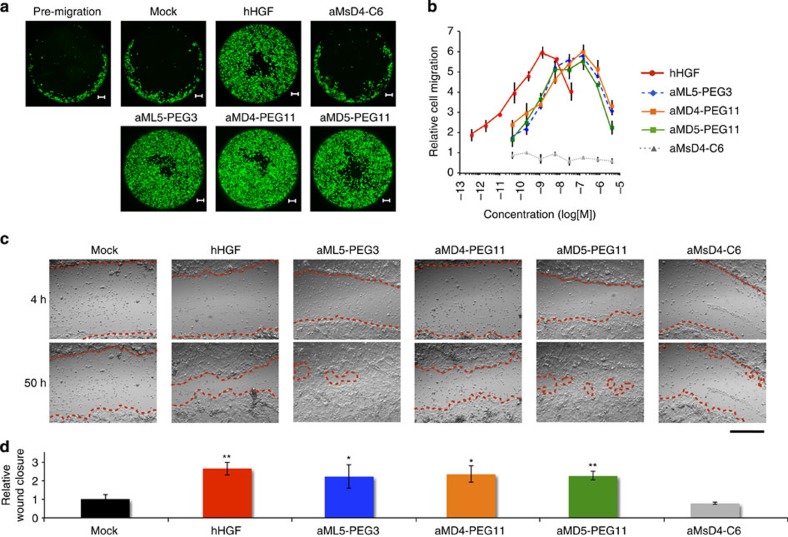
Cellular migration and wound healing promoted by dimeric macrocycles. (**a**) Captured images for migration of normal human RPTEC stimulated by hHGF (0.26 nM) or dimer macrocycles (20 nM) over 30 h. Cells were stained by calcein-AM. Scale bar, 200 μm. (**b**) Dose-dependent titration of cell migration stimulated by 0.4 pM–30 nM of hHGF (red), 40 pM–3,000 nM of aML5-PEG3 (blue), aMD4-PEG11 (orange), aMsD4-C6 (grey) as a negative control, or aMD5-PEG11 (green). Migrated cells were stained with calcein-AM and quantified by fluorescence intensity. s.d. was calculated from the results of experiments in triplicate. (**c**) Captured images of wound healing in NHEK promoted by various stimulants (also see Supplementary Movie 1). Wound-closure events in the presence or the absence of 0.25 nM hHGF or 100 nM dimeric macrocycles were monitored by a real-time cultured cell monitoring system. The images were taken at 4 and 50 h. Red broken lines indicate boundaries between cells in the monolayer and the scratched areas uncovered by cells. Scale bar, 500 μm. (**d**) Quantification of relative wound-closure areas. Error bars denote s.e.m. (*n*=3). *P<0.05, **P<0.01 (unpaired Student’s *t*-test) compared with Mock.

Cell migration stimulated by hHGF is also a critical phenotypic event in embryonic development^28^ and wound healing^29^. Therefore, we tested whether the dimeric macrocycles could promote migration of RPTEC. Each macrocycle dimer induced cell migration in a dose-dependent manner, with observed ED_50_ in the range of 1–3 nM (Fig. 5a,b). On the other hand, the inactive aMsD4-C6 did not induce migration. The maximal activity of the dimeric macrocycles was observed at around 100 nM. Notably, bell-shaped dose-dependent profiles were again observed in these experiments, consistent with the aforementioned dose–response studies.

As the HGF-Met receptor pathway is essential for cutaneous wound healing and re-epithelialization^30^, we next performed a wound-healing assay, using NHEK cells (Fig. 5c,d and Supplementary Movie 1). A monolayer culture of NHEK cells was scratched and cultured in the absence or presence of the dimeric macrocycles or hHGF for 50 h. Treatment with the Met-inactive aMsD4-C6 resulted in only marginal closure of the scratched area, comparable to an unstimulated control. In contrast, the dimeric macrocycles strongly promoted closure of the scratched area with effects comparable to hHGF.

**Figure 6:**
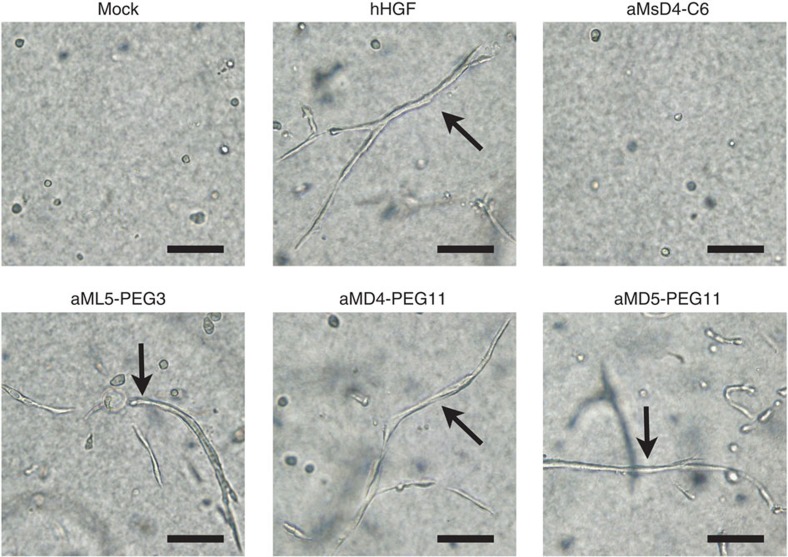
Branching morphogenesis in normal human RPTEC, induced by dimeric macrocycles. RPTEC encapsulated in collagen gels were cultured in the presence of 0.42 nM hHGF or 30 nM dimeric macrocycles over 8 days. Arrows indicate tubules formed by RPTEC. Scale bars, 100 μm.

As hHGF is known to induce the branching morphogenesis^22^,^26^,^31^ that builds epithelial architectures during embryogenesis, we finally examined whether the dimeric macrocycles could stimulate the formation of tubules in a population of RPTEC. The cells were encapsulated in collagen gels and soaked in a culture medium containing the dimeric macrocycles. After 8 days, cultures exposed to hHGF or dimeric macrocycles developed branched tubules (Fig. 6 and Supplementary Fig. 15a). Such tubules were absent in cultures exposed to aMsD4-C6 as a negative control. The same branching morphogenesis promoted by the dimeric macrocycles was also observed for HuCCT1 cells (Supplementary Fig. 15b). In summary, the dimeric macrocycles described in these studies induce the same phenotypic events as hHGF in every assay tested.

## Discussion

The chemical cross-linking experiments indicate that the cell-surface Met dimerization triggered by both the dimeric macrocycles and HGF showed bell-shaped dose-dependent profiles (Fig. 3e and Supplementary Fig. 9). As the dimerization of Met is a critical initial event for intracellular tyrosine transphosphorylation, our data indicate that the bell-shaped responses in phosphorylation of Met (Fig. 3b–d), cellular proliferation (Fig. 4a–c) and migration (Fig. 5b–d) can be primarily attributed to the efficiency of Met dimerization. The bell-shaped Met dimerization profile could be explained by a theoretical ‘receptor-cross-linking model’ proposed for receptors with bivalent ligands bearing two chemically identical functional groups^32^. The model indicates receptor–ligand stoichiometry as follows: following a peak in the formation of dimerized receptor–ligand complex, R_2_L (2:1 complex of R/receptor and L/ligand), an excess concentration of ligand would increase the relative proportion of inactive non-dimerized receptor–ligand complex, RL (1:1 complex of R and L), while the proportion of active R_2_L complex would decline. Therefore, diminution of R_2_L caused by exhaustion of R through conversion to RL at an excess amount of ligand (that is, dimeric macrocycles in our case) could be a reason for the bell-shaped responses. On the other hand, a 2:2 complex between HGF and Met in which the dimerization interface is provided by two HGF molecules has been proposed for Met activation^6^. Given such a model, hHGF may form an inactive RL_2_ complex rather than an active R_2_L_2_ at concentrations of hHGF in excess of the optimum for R_2_L_2_ formation.

Using the RaPID system, we have generated macrocycles that strongly bind to Met with remarkably high specificity. Dimerization of the macrocycles turned them into potent agonists capable of specifically activating the Met signalling pathway (Fig. 3). Consistent with the phosphorylation of Met and the subsequent signalling proteins, the dimeric macrocycles are able to clearly mimic the functional role of hHGF and induce cellular responses in not only model cell lines but also various normal human cells (Figs 4, 5, 6 and Supplementary Figs 13 and 14). As the observed branching morphogenesis would be expected to require coordinated proliferation and migration of cells, the dimeric macrocycles could fulfill the function of regenerative drugs for the repair of tissue damage or for other therapeutic uses. In addition, as recombinant hHGF has been used for the differentiation of induced pluripotent stem cells into hepatocytes^33^, an appropriate replacement of hHGF with the dimeric macrocycles that are chemically synthesized assures no biological contaminants of organismal origin. Thus, our dimeric macrocycles are applicable to tissue engineering and regenerative medicine applications using such stem cells.

To the best of our knowledge, the macrocycles reported here represent the most potent artificial agonists of Met ever made and demonstrate the generation of artificial ‘non-protein’ growth factors structurally unrelated to the original protein. Replacement of proteins by the artificial small-sized dimeric macrocycles seems to have advantages for drug development, such as production by chemical synthesis and amenability to chemical modifications for the control of pharmacokinetics, protease resistance and drug delivery, and such controlled properties should be carefully evaluated in future research and development. Despite the fact that the dimeric macrocycles tightly bind to the ectodomain of human Met and induce various cellular responses in human cells, they did not show any binding activity to that of murine or canine Met (Supplementary Table 3 and Supplementary Fig. 4). We also tested the influence of the dimeric macrocycles to cellular responses on murine and canine cell lines (Supplementary Table 5 and Supplementary Fig. 16) but no effect was observed, confirming that the dimeric macrocycles are specific to human Met. The above observation suggests that a hominid species or a modified mouse carrying the human *Met* gene seems essential, to examine the human therapeutic efficacy of these dimeric macrocycles in future animal experiments.

Most importantly, the RaPID selection can be applied to various membrane proteins^34^,^35^,^36^,^37^, including other RTKs, so that we will be able to obtain macrocycles that specifically bind such designated targets. As many of these transmembrane receptors are dimerized or heterodimerized by the interaction with cognate ligands to activate signalling pathways^1^,^38^, the methodology reported here is applicable to the design and synthesis of such artificial agonists.

## Methods

### Materials

Recombinant human Met ectodomain-Fc, murine Met ectodomain-Fc chimera proteins and canine Met ectodomain were purchased from Sino Biological Inc. Recombinant human Fc protein and human recombinant EGF were purchased from R&D Systems. hHGF was biotinylated using NH_2_-Reactive Biotin (Dojindo). The molar ratio was 1.5 biotin per hHGF as determined using a Biotin Quantitation Kit (Pierce). The Met phosphorylation activity of biotinylated hHGF was equivalent to that of hHGF as evaluated by cell-based phospho-Met ELISA. NHS (*N*-hydroxysuccinimide)-fluorescein, NHS-biotin, NHS-PEO4-biotin and bismaleimidohexane were purchased from Thermo Scientific. Bis-MAL-dPEG3 (BMPEG3) and Bis-MAL-dPEG11 (BMPEG11) linkers were purchased from Quanta BioDesign. The following antibodies were purchased from Cell Signaling Technology: Met (25H2) mouse mAb, phospho-Met (Tyr1234/1235) (D26) XP rabbit mAb, Akt (11E7) rabbit mAb, phospho-Akt (Ser473) (D9E) XP rabbit mAb, Erk1/2 (137F5) rabbit mAb, phospho-Erk1/2 (Thr202/Tyr204) (D13.14.4E) XP rabbit mAb, Gab1 rabbit mAb, phospho-Gab1 (Tyr 627) (C32H2) rabbit mAb and phospho-EGFR (Tyr 1068) rabbit polyclonal Ab. Anti-phospho-Met (Tyr1349) (07-808) rabbit polyclonal antibody was purchased from Merck Millipore. Anti-phosphorylated c-Ret (Y905) rabbit polyclonal antibody was purchased from R&D Systems. Anti-human Met rat mAb for live-cell imaging was purchased from eBioscience. Alexa Fluor 488 goat anti-rabbit IgG (HL) and Alexa Fluor 594 goat anti-rat IgG (HL) were purchased from Life Technologies. Horseradish peroxidase (HRP)-conjugated anti-rabbit goat antibody was purchased from Dako.

### Selection of anti-Met macrocycles

The random mRNA library and the two non-canonical aminoacyl-transfer RNAs, ClAc-L-Tyr-tRNA^fMet^_CAU_ and ClAc-D-Tyr-tRNA^fMet^_CAU_ were prepared as previously reported^39^. Briefly, 40 μM tRNA^fMet^_CAU_, 600 mM MgCl_2_ and 5 mM ClAc-L-Tyr-CME or ClAc-D-Tyr-CME in dimethylsulfoxide (DMSO) were mixed in 100 mM HEPES-KOH (pH 8.0) and incubated on ice for 1 h. After the reaction, one-tenth of a volume of 3 M sodium acetate (pH 5.2) was added and the RNA was ethanol precipitated. The pellet was rinsed with 70% (v/v) ethanol containing 0.1 M sodium acetate (pH 5.2), then 70% ethanol only. The pellet was air dried and dissolved in 1 mM sodium acetate (pH 5.2) before use. Thioether macrocycles targeting human Met ectodomain were selected using the RaPID system^13^,^39^, slightly modified as follows: 1 μM mRNA library was incubated with 1.5 μM puromycin linker in the presence of T4 RNA ligase for 30 min at 25 °C and was purified by phenol–chloroform extraction and ethanol precipitation. To generate the respective peptide library initiated with ClAc-L-Tyr (L-library) or ClAc-D-Tyr (D-library), 1.4 μM mRNA–puromycin library was translated in a methionine-deficient FIT system^14^ at a scale of 150 μl total volume in the presence of 50 μM ClAc-L-Tyr-tRNA^fMet^_CAU_ or ClAc-D-Tyr-tRNA^fMet^_CAU_ for 30 min at 37 °C. After a 12-min incubation at 25 °C, the temperature was elevated to 37 °C and maintained for 30 min, to promote macrocyclization. The fused macrocycle–mRNA was subsequently reverse transcribed by RQ-RTase (Promega) for 1 h at 42 °C and incubated with human recombinant Fc protein-immobilized Dynabeads protein G (Life Technologies) for 30 min at 4 °C (negative selection against Fc-tag and beads). The unbound fraction was then incubated with human Met ectodomain-Fc chimera protein immobilized on Dynabeads protein G for 30 min at 37 °C. The resultant complementary DNAs were eluted by mixing with 1 × PCR reaction buffer and heating at 95 °C for 5 min, followed by immediate separation of the supernatant from the beads. A small fraction of the cDNA was allocated to real-time PCR quantification using a LightCycler 2.0 (Roche); the remainder was amplified by PCR. The resulting duplex DNAs were purified by phenol–chloroform extraction and ethanol precipitation, and transcribed into mRNAs for the next round of selection. In the second round, the translation was performed at 5 μl scale, and from the third to fifth rounds the scale was lowered to 2.5 μl. The isolated cDNAs from the pool enriched in the fourth and fifth rounds were ligated into the pGEM-T-Easy vector (Promega), using DNA Ligation Kit Mighty Mix (Takara Bio), and cloned into DH5α competent cells. The cloned DNAs were then sequenced.

### Chemical synthesis of macrocycles

Macrocycles were synthesized by standard Fmoc solid-phase peptide synthesis (SPPS) using a Syro Wave automated peptide synthesizer (Biotage). The resulting peptide–resin (25 μmol scale) was treated with a solution of 92.5% trifluoroacetic acid (TFA), 2.5% water, 2.5% triisopropylsilane and 2.5% ethanedithiol, to yield the free linear *N*-ClAc-peptide. Following diethyl ether precipitation, the pellet was dissolved in 10 ml triethylamine containing DMSO and incubated for 1 h at 25 °C, to yield the corresponding macrocycle. The peptide suspensions were then acidified by addition of TFA to quench the macrocyclization reaction. The macrocycle was purified by reversed-phase HPLC (RP-HPLC), using a Prominence HPLC system (Shimadzu) under linear gradient conditions. Mobile phase A (comprising water with 0.1% TFA) was mixed with mobile phase B (0.1% TFA in acetonitrile). Purified peptides were lyophilized *in vacuo* and molecular mass was confirmed by matrix-assisted laser desorption/ionization time-of-flight MS, using an AutoFlex II instrument (Bruker Daltonics).

### C-terminal modification of macrocycles

For fluorescein- or biotin-labelling of aMD5, aMD5-Lys(Mmt)-NH-resin was synthesized by Fmoc SPPS and the Mmt group was then deprotected using 98% dichloromethane, 1% TFA and 1% triisopropylsilane. The resulting aMD5-Lys-NH-resin was equilibrated with 20% *N*,*N*-diisopropylethylamine in *N*-methylpyrrolidone and treated with 0.2 M NHS-fluorescein or NHS-biotin in *N*,*N*-diisopropylethylamine in *N*-methylpyrrolidone. The modified peptide was deprotected/cleaved and macrocyclized as described above, followed by RP-HPLC purification and *in vacuo* lyophilization. For the same labelling of aML5, aMD4 and aMsD4, the respective peptide-Lys was synthesized by the Fmoc SPPS, deprotected/cleaved and macrocyclized as above. Following RP-HPLC purification, a 20-mM solution of each macrocycle in DMSO was treated with 0.2 M of NHS-fluorescein, NHS-biotin or NHS-PEO4-biotin, and then purified by RP-HPLC. To synthesize the dimeric macrocycles, a 22-mM solution of the respective C-terminal cysteine-modified monomer peptide in DMSO was incubated with 10 mM of the bis-maleimide linker in 50 mM Hepes-HCl (pH 7.5) with 90% (v/v) DMSO. The resulting dimer was purified by RP-HPLC and lyophilized *in vacuo*.

### SPR analysis of macrocycle

The binding constants of the macrocycles to the Met ectodomain were determined by SPR analysis using a Biacore T200 machine (GE Healthcare). The running buffer was HBS EP+ buffer (10 mM Hepes pH 7.4, 150 mM NaCl, 3 mM EDTA and 0.05% (v/v) SurfactantP20) containing 0.1% DMSO. To assess the interaction between human Met and the macrocycles, human Met ectodomain-Fc chimera was immobilized to a surface density of 1,200–1,600 response units (RUs) on a CM5 sensor chip using a human antibody capture kit (GE Healthcare). Macrocycle binding was tested by injecting varying concentrations of the macrocycle at a flow rate of 30 μl min^−1^. Binding was quantified by a single-cycle kinetics method. To assess whether macrocycles and hHGF interacted with human, mouse and canine Met, each biotinylated macrocycle or hHGF was immobilized to a surface density of 200–500 RUs (for biotinylated macrocycles) or ~2,200 (for biotinylated hHGF) RUs on a CAP sensor chip using a Biotin CAPture Kit (GE Healthcare), following the standard protocol. Met binding was tested by injecting varying concentrations of the macrocycle at 30 μl min^−1^ and quantifying by a single-cycle kinetics method. All data were fitted to the standard 1:1 binding model.

### Immunoprecipitation assay using FLAG-tagged macrocycles

Proteins bound to FLAG-tagged macrocycles were immunoprecipitated and identified as previously reported^17^. Briefly, HepG2 cells were incubated with 200 nM FLAG-tagged macrocycle for 1 h. After washing with PBS, cells were lysed with lysis buffer containing 1% Triton X-100. The lysate was immunoprecipitated by FLAG-M2 agarose (Sigma-Aldrich). Immunoprecipitates were digested by Lys-C endopeptidase and analysed by direct nano-flow LC–MS/MS. Experiments were performed in quadruplicate.

### Cell culture

The EHMES-1 human mesothelioma cell line was supplied by Dr Hamada (Ehime University, Japan). The HuCCT1 human bile duct carcinoma cell line was obtained from the Japanese Cancer Research Resources Bank. The TAC-2 mouse mammary epithelial cell line and MDCK canine kidney epithelial cell line were provided by Dr Montesano (University of Geneva, Switzerland). The HCC827, gefitinib-sensitive human lung adenocarcinoma cell line was obtained from the American Type Culture Collection (ATCC). Normal HUVEC, HDMEC, HPAEC and NHEK cells were obtained from PromoCell. RPTEC was obtained from ATCC. EHMES-1, HuCCT1 and HCC827 cells were maintained in RPMI1640 medium supplemented with 10% (v/v) FBS. TAC-2 and MDCK cells were maintained in DMEM supplemented with 10% FBS. HUVEC and HDMEC were maintained in Endothelial Cell Basal Medium (PromoCell) supplemented with 2% FCS, 0.4% endothelial cell growth supplement, 0.1 ng ml^−1^ recombinant human EGF (rh EGF), 1 ng ml^−1^ recombinant human basic fibroblast growth factor, 90 μg ml^−1^ heparin and 1 μg ml^−1^ hydrocortisone. NHEK cells were maintained in Keratinocyte Basal Medium 2 (KBM2, PromoCell) supplemented with 0.4% bovine pituitary extract, 0.125 ng ml^−1^ rh EGF, 5 μg ml^−1^ rh insulin, 0.33 μg ml^−1^ hydrocortisone, 0.39 μg ml^−1^ epinephrine, 10 μg ml^−1^ transferrin and 0.06 mM CaCl_2_. RPTEC were maintained in Renal Epithelial Cell Basal Medium (REBM, ATCC) supplemented with 0.5% FBS, 10 nM triiodothyronine, 10 ng ml^−1^ rh EGF, 100 ng ml^−1^ hydrocortisone hemisuccinate, 5 μg ml^−1^ rh insulin, 1 μM epinephrine, 5 μg ml^−1^ transferrin and 2.4 mM L-alanyl-L-glutamine.

### Live-cell imaging using fluorescently labelled macrocycles

EHMES-1 cells (2 × 10^4^ cell per well) were seeded in a 96-well glass bottom plate and cultured for 24 h at 37 °C. For Met overexpression, cells were transfected with pCAGGS-MET plasmid using FuGENE HD (Promega) 48 h before observation. Cells were then incubated in RPMI1640 medium containing 5 μM fluorescein-labelled macrocycle, 1 μg ml^−1^ anti-human Met rat mAb and 1 μg ml^−1^ Hoechst 33342 (Dojindo) for 5 min at 4 °C. Cells were washed three times with RPMI1640 medium and incubated with 1 μg ml^−1^ Alexa Fluor 594 goat anti-rat IgG (HL) for 5 min at 4 °C. Next, the cells were rewashed three times with fresh RPMI1640 medium and observed by fluorescent microscopy (F6000, Leica Microsystems).

### Cell-based phospho-Met ELISA

EHMES-1 cells (2 × 10^4^ cells per well) were seeded in a 96-well black μClear-plate (Greiner Bio-One). After 24 h, the cells were stimulated with hHGF or dimeric macrocycle in RPMI1640 medium supplemented with 10% FBS for 10 min. The cells were washed with ice-cold PBS, fixed with 4% paraformaldehyde (PFA) in PBS for 30 min and washed three times with PBS. The cells were blocked with 5% goat serum, 0.02% Triton X-100 in PBS for 30 min and then incubated for 12 h in phospho-Met (Tyr1234/1235) (D26) XP rabbit mAb diluted 1:1,000 in 1% goat serum in PBS at 4 °C. The cells were washed three times with PBS and incubated for 1 h in HRP-conjugated anti-rabbit goat antibody diluted 1:1,000 in 1% goat serum in PBS. Following incubation, the cells were washed four times with PBS. Chemiluminescence was developed with ImmunoStar LD reagent (Wako) and measured by ARVO MX (Perkin Elmer). Relative Met phosphorylation was calculated as (Chemiluminescence unit of sample−chemiluminescence unit of mock control)/(chemiluminescence unit of 1.3 nM hHGF−chemiluminescence unit of mock control).

### Western blotting and phospho-RTK array

EHMES-1 cells were cultured in a six-well plate until they were 80%–90% confluent. After starvation for 6 h, the cells were stimulated with 2 nM hHGF or with 100 nM aML5-PEG3, aMD4-PEG11, aMD5-PEG11 or aMsD4-C6 for 10 min. Cells were then lysed with 200 μl of lysis buffer 17 (R&D Systems) containing 1 × Complete protease inhibitor cocktail (Roche). Lysates were analysed by SDS–PAGE analysis with 10% polyacrylamide gel and standard western blotting. The respective primary antibodies and HRP-conjugated secondary antibodies were diluted 1:2,000 in Can Get Signal Solution 1 (TOYOBO) and 1:5,000 in Can Get Signal Solution 2 (TOYOBO), respectively. Chemiluminescent signals were developed with Luminata Forte HRP substrate (Merck Millipore) and observed using an ImageQuant LAS 350 machine (GE Healthcare). Relative phosphorylation efficiency was evaluated by the band intensity and statistical differences between stimulants were calculated using Student’s *t*-test. The phospho-RTK array was performed using Proteome Profiler Human Phospho-RTK Array Kit (R&D Systems). For stimulation of cells with FBS, cells were cultured in the presence of 10% FBS, with or without excess concentrations of hHGF (30 nM) or aML5-PEG3 (3,000 nM) for 10 min. Cell lysates of stimulated EHMES-1 cells were analysed according to the manufacturer’s instructions.

### EGF-induced phosphorylation of EGFR

EHMES-1 cells were cultured in a 12-well plate until they were 80%–90% confluent. After starvation for 6 h, the cells were left untreated or stimulated with 0.2 nM human recombinant EGF in the presence or absence of the indicated concentrations of gefitinib or aML5-PEG3 for 5 min. Cells were then lysed with 100 μl of Laemmli sample buffer. Lysates were sonicated and centrifuged for 15 min at 15,000 *g*. Supernatants were analysed by SDS–PAGE gel electrophoresis and western blotting, using anti-phosphorylated EGFR (Y1068 1:1,000 dilution) rabbit polyclonal antibody or anti-phosphorylated Met (Y1234/1235, 1:1,000 dilution) (D26) rabbit mAb.

### Transactivation of c-Ret

EHMES-1 cells were cultured in a 12-well plate until they were 80%–90% confluent. The cells were left untreated or stimulated with hHGF or aML5-PEG3 in the presence or absence of the indicated concentrations of the specific Met kinase inhibitor, SU11274 (Calbiochem). Cells were then lysed with 100 μl of Laemmli sample buffer. Lysates were sonicated and centrifuged for 15 min at 15,000 *g*. Supernatants were analysed by SDS–PAGE gel electrophoresis and western blotting, using anti-phosphorylated Met (Y1234/1235) mAb or anti-phosphorylated c-Ret (Y905) antibody.

### Immunofluorescence analysis of phosphorylated Met on cells

EHMES-1 cells (2 × 10^4^ cells per well) were seeded in a 96-well glass bottom plate and cultured in RPMI1640 medium supplemented with 10% FBS for 24 h. After starving for 6 h, cells were stimulated with 2 nM of hHGF, 100 nM of aML5-PEG3, aMD4-PEG11, aMD5-PEG11 or aMsD4-C6 for 10 min. Stimulated cells were washed with PBS and fixed with 4% PFA/PBS containing 0.1% Triton X-100 at 25 °C for 15 min. After washing with TBST (50 mM Tris-HCl, 150 mM NaCl, 0.05% Tween-20 and pH 7.6), the fixed cells were blocked using Blocking One reagent (Nacalai Tesque) containing 0.1% Triton X-100 for 1 h at 25 °C. Cells were washed three times with TBST and incubated in a mixture of two primary antibodies: 1 μg ml^−1^ anti-human Met rat mAb and phospho-Met (Tyr1234/1235) (D26) XP rabbit mAb diluted by 1:1,000 in Can Get Signal Solution 1 for 1 h at 25 °C. After washing with TBST, cells were stained in a mixture of 1 μg ml^−1^ Alexa Fluor 594 goat anti-rat IgG (HL), 1 μg ml^−1^ Alexa Fluor 488 goat anti-rabbit IgG (HL) and Hoechst 33342 in Can Get Signal Solution 2 for 30 min at 25 °C. Cells were washed three times with TBST and observed using an F6000 fluorescent microscopy system.

### Analysis of Met dimerization by cross-linking of live cells

EHMES-1 cells were cultured in a six-well plate until they were 80%–90% confluent. Cells were washed twice with ice-cold RPMI1640 medium with 10% FBS and incubated for 1 h at 4 °C, with the indicated concentrations of dimer macrocycles or hHGF in RPMI1640 medium containing 10% FBS. Cells were then washed three times with ice-cold PBS and incubated for 1 h at 4 °C with 1 mM bis(sulfosuccinimidyl) suberate (BS3, non-cell permeable cross-linker, Thermo Scientific) in PBS. Non-reactive BS3 was quenched with 50 mM Tris (pH 8.0), 150 mM NaCl for 15 min at 4 °C. Cells were washed three times with ice-cold PBS and lysed in lysis buffer (40 mM Tris-HCl (pH 8.0), 1% Triton X-100, 1% NP-40, 10% glycerol, 0.15 M NaCl, 2 mM EDTA, 1 mM phenylmethanesulfonyl fluoride, 1 × Complete protease inhibitor cocktail). Cell lysates were passed through 27-G needle and the extracts were centrifuged for 15 min at 15,000 *g*. Supernatants were incubated for 4 h at 4 °C with 1 μg anti-Met rabbit polyclonal antibody (Santa Cruz), then incubated for 12 h at 4 °C with 30 μl of Dynabeads protein G (Invitrogen). The immunoprecipitates were washed three times with lysis buffer and were eluted with SDS–PAGE sample buffer under reducing conditions. The samples were analysed by 5–20% PAGE and western blotting was performed using an anti-Met rabbit mAb (D1C2, Cell Signaling Technology).

### Cell growth assay

Cells were plated at 2,500 cells per well in 96-well plates for calcein-AM staining or 7,500 cells per well in 48-well plates for cell counting. For HUVEC and HDMEC, plates were pre-coated with gelatin (Difco Laboratories, 0.1% for 1 h at 37 °C). After 3 h, the medium was removed and replaced with medium as follows: HUVEC and HDMEC were cultured in endothelial cell basal medium supplemented with 2% (HUVEC) or 1% (HDMEC) FCS, and 90 μg ml^−1^ heparin, with or without hHGF or dimeric macrocycle. RPTEC were cultured in REBM supplemented with 0.5% FBS and 2.4 mM L-alanyl-L-glutamine, with or without hHGF or dimeric macrocycle. NHEK was cultured in KBM2 supplemented with 0.4% bovine pituitary extract, 0.33 μg ml^−1^ hydrocortisone, 0.39 μg ml^−1^ epinephrine, 10 μg ml^−1^ transferrin and 1.8 mM CaCl_2_, with or without hHGF or dimeric macrocycle. HCC827 cells were cultured in RPMI1640 supplemented with 5% FBS and 1 μM gefitinib, with or without hHGF or dimeric macrocycle. The culture media (with or without hHGF or dimeric macrocycle) were replaced every second day. After 6 days, cells were stained with 5 μg ml^−1^ calcein-AM (Dojindo) at 37 °C for 15 min. Fluorescence of living cells was detected by ARVO MX. Alternatively, cells were harvested with trypsin–EDTA solution, suspended in culture medium and the cell number was determined using a Countess Automated Cell Counter (Life Technologies). Statistical differences between stimulants were calculated using Student’s *t*-test.

### DNA synthesis

Normal HPAECs were maintained in MCDB 131 medium supplemented with 5% FBS and 1.2 nM of basic fibroblast growth factor. Cells were serum starved for 24 h and then stimulated with 30 nM dimeric macrocycle or 0.25 nM hHGF in the presence of 5% FBS and 10 μM BrdU for 20 h. The cells were fixed in 4% PFA in PBS for 30 min, permeabilized with 0.2% Triton X-100 in PBS for 5 min, treated with 2 M HCl for 20 min and neutralized with 0.1 M sodium tetraborate for 5 min. The treated, neutralized cells were incubated with anti-BrdU primary antibody (BD Biosciences) for 2 h and subsequently stained with a fluorescently labelled secondary antibody for 30 min. The nuclei were stained with DAPI. BrdU incorporation was analysed by Biozero fluorescence microscopy (Keyence).

### Cell migration assay

Cell migration was assayed by Oris Cell Migration Assays (Platypus Technologies) or Transwell Migration Assay. To prepare Oris Cell Migration Assays, RPTEC (6 × 10^4^ cells per well) were cultured for 24 h in REBM supplemented with 0.5% FBS, 2.4 mM L-alanyl-L-glutamine and 1.8 mM CaCl_2_. Stoppers were removed, cells were washed twice and cultured in REBM supplemented with 0.5% FBS, 2.4 mM L-alanyl-L-glutamine and 1.8 mM CaCl_2_, with or without hHGF or dimeric macrocycle, and allowed to migrate into the migration zone. After culturing for 30 h, cells were stained with 5 μg ml^−1^ calcein-AM at 37 °C for 15 min. Fluorescence of migrated cells was detected by ARVO MX. Cell images were captured by BIOREVO BZ-9000 (KEYENCE). To prepare the Transwell assay, murine melanoma cells (1 × 10^5^ cells per insert) cultured in 200 μl RPMI1640 medium supplemented with 0.5% FBS were plated on the upper insert (6.5 mm diameter transwell with 8 μm pores, Corning), while 800 μl RPMI1640 medium supplemented with 0.5% FBS, with or without hHGF or dimeric macrocycle was added to the bottom chamber. Cells were cultured for 16 h and fixed with 4% PFA in PBS. Cells attached to the bottom side of the membranes were stained with 0.4% crystal violet in 20% methanol.

### Wound-healing assay

NHEK (4 × 10^5^ cells) were plated into 96-well plates and incubated in KBM2 supplemented with 0.4% bovine pituitary extract, 0.125 ng ml^−1^ rh EGF, 5 μg ml^−1^ rh insulin, 0.33 μg ml^−1^ hydrocortisone, 0.39 μg ml^−1^ epinephrine, 10 μg ml^−1^ transferrin and 0.06 mM CaCl_2_ for 24 h, to allow the formation of a confluent monolayer. The monolayer was wounded with a 1,000-μl pipette tip (Watson), washed with media and incubated in growth medium without rh EGF and with 0.25 nM hHGF or 100 nM dimeric macrocycles for 50 h. Cell migration was monitored in real time using a cultured cell monitoring system (CCM-1.4XYZ/CO2, Astec Co.) and wound closure area was measured using CCM software. Movies were generated using ASBRO software (Astec Co.). Statistical differences between stimulants were calculated with Student’s *t*-test.

### Branching morphogenesis assay

RPTEC (2 × 10^4^ cells) were suspended in 400 μl Cellmatrix type IA collagen solution (Nitta Gelatin) in a 48-well plate and allowed to gel for 60 min at 37 °C, according to the manufacturer’s instructions. After gelation, 500 μl REBM supplemented with 0.5% FBS and 2.4 mM L-alanyl-L-glutamine, with or without hHGF (0.42 nM, 1.3 nM) or macrocycles (30 nM, 100 nM), were added to each well. Cells were cultured for 8 days, replacing the culture medium (with or without hHGF or dimeric macrocycle) every second day. HuCCT1 (1 × 10^4^ cells) were suspended in 400 μl Cellmatrix type IA collagen solution in a 48-well plate and were allowed to gel for 60 min at 37 °C. After gelation, 500 μl RPMI1640 medium supplemented with 10% FBS, with or without 0.38 nM hHGF or 20 nM dimeric macrocycle, was added to each well. Cells were cultured for 8 days, replacing the culture medium (with or without hHGF or dimeric macrocycle) every second day. TAC-2 cells (4 × 10^3^ cells) were suspended in 400 μl type I collagen solution (BD Biosciences) in a 48-well plate. After gelation, 500 μl DMEM supplemented with 10% FBS, with or without hHGF or dimeric macrocycle, was added to each well. Cells were cultured for 7 days, replacing the culture medium (with or without hHGF or dimeric macrocycle) every second day.

### Scatter assay

MDCK cells (1 × 10^4^ cells) were seeded in 200 μl DMEM with 10% FBS in a 48-well plate and cultured for 12 h. Cells were stimulated with hHGF or dimeric macrocycles in DMEM supplemented with 10% FBS and cultured for a further 12 h. The stimulated cells were fixed with 4% PFA in PBS for 30 min and stained with 0.2% crystal violet in 20% methanol.

## Author contributions

K.I. developed the methodology and performed the selection and biochemical experiments. K.S., Y.S. and K.M. designed and performed the biological assays. N.O. contributed to data analysis of bell-shaped profiles, and T.H. and T.N. conducted proteomics analysis. K.I., K.S. and K.M. contributed to writing the manuscript. H.S. directed the programme and wrote the manuscript.

## Additional information

**How to cite this article:** Ito, K. *et al*. Artificial human Met agonists based on macrocycle scaffolds. *Nat. Commun.* 6:6373 doi: 10.1038/ncomms7373 (2015).

## Supplementary Material

Supplementary Information Supplementary Figures 1-18 and Supplementary Tables 1-5.

Supplementary Movie 1 Wound closure of normal human epidermal keratinocytes. Cells with scratch wounds were stimulated by 0.25 nM hHGF or 100 nM dimeric macrocycles over 50 h to promote the wound healing activity.

## Figures and Tables

**Figure 1:**
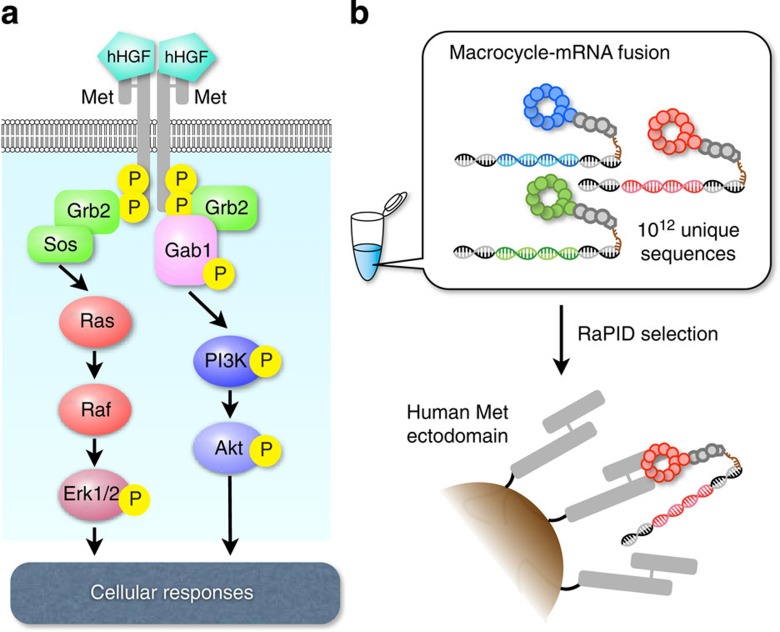
The human Met signalling cascade and RaPID selection. (**a**) When human HGF (hHGF) binds to the Met ectodomain, it forms a 2:2 dimerization complex, bringing intracellular tyrosine kinase domains into close proximity and inducing *trans*-phosphorylation. Met activation transduces major signals through the adaptor protein Gab1. Activation of downstream cytoplasmic kinases such as Akt and Erk1/2 (mitogen-activated protein kinase (MAPK)) eventually promote cellular phenotypic changes. (**b**) Using the RaPID system, we can select artificial macrocycles fused to the respective mRNAs against a target: in this case, the human Met ectodomain. During the selection process, binding species are enriched from an initial library of 10^12^ unique members of macrocycle–mRNA fusions. PCR amplification of cDNAs and cDNA transcription and translation can be repeated until the active species dominates (Supplementary Fig. 1).
