# Supplementary material for: Artificial human Met agonists based on macrocycle scaffolds
Source: Nat Commun. 2015 Mar 11;6:6373. doi: 10.1038/ncomms7373 (PMC4382702; doi:10.1038/ncomms7373)
Supplement: Supplementary Information — Supplementary Figures 1-18 and Supplementary Tables 1-5. [file ncomms7373-s1.pdf]

Supplementary Figure 1

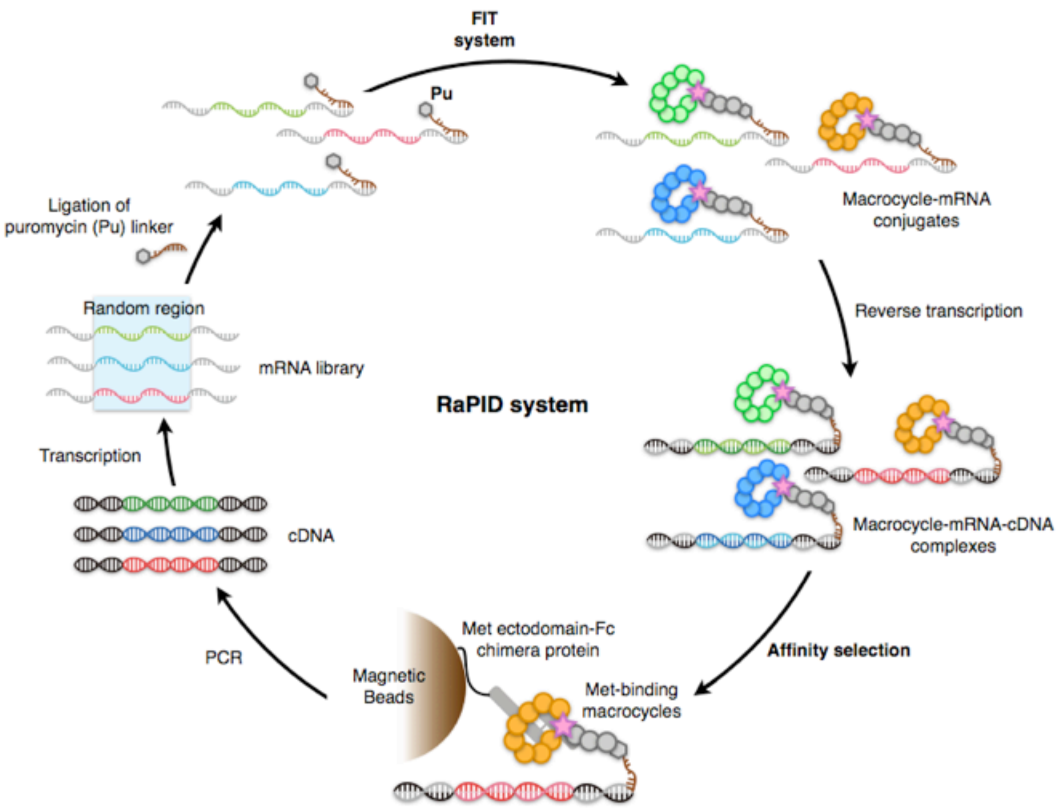

Supplementary Figure 1. Schematic of the RaPID system for development of human Met ectodomain-binding macrocycles. First, an mRNA library containing random regions was ligated with puromycin (Pu) linkers. Ligation products were *in vitro* translated in the FIT system, and the translated *N*-ClAc-peptides were spontaneously macrocyclized to obtain random thioether macrocycles. Macrocycle-mRNA conjugates were reverse-transcribed into macrocycle-mRNA-cDNA. Affinity selection was performed against human Met ectodomain-Fc chimera protein immobilized on magnetic beads. The cDNAs encoding Met-binding macrocycles were recovered and amplified by PCR. Amplified cDNAs were transcribed into mRNAs for the next selection round. The selection cycle was repeated until Met-binding macrocycles were enriched.

Supplementary Figure 2

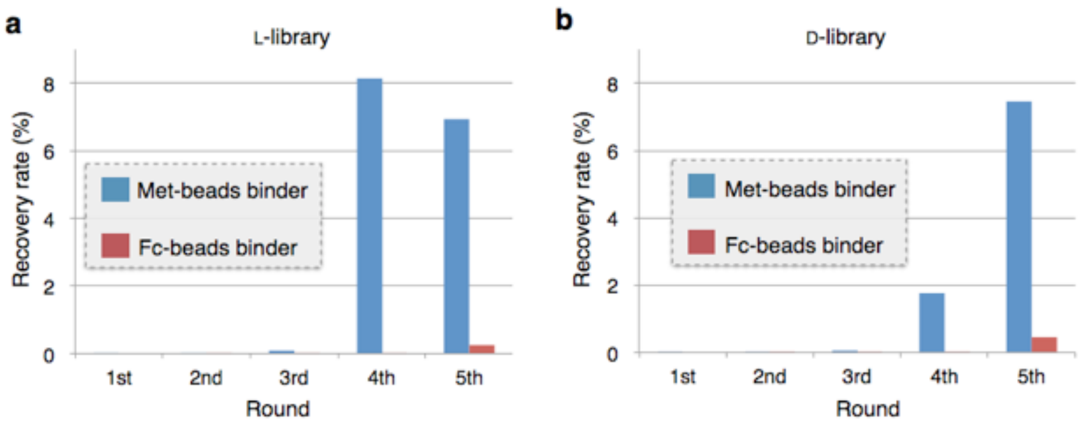

Supplementary Figure 2. Progress of selections from (**a**) L- and (**b**) D-libraries. Blue and red bars represent the recovery rates of the cDNAs eluted from macrocycle-mRNA complexes binding to human Met ectodomain-Fc chimera protein-immobilized magnetic beads (Met-beads) or Fc-immobilized magnetic beads (Fc-beads).

Supplementary Figure 3

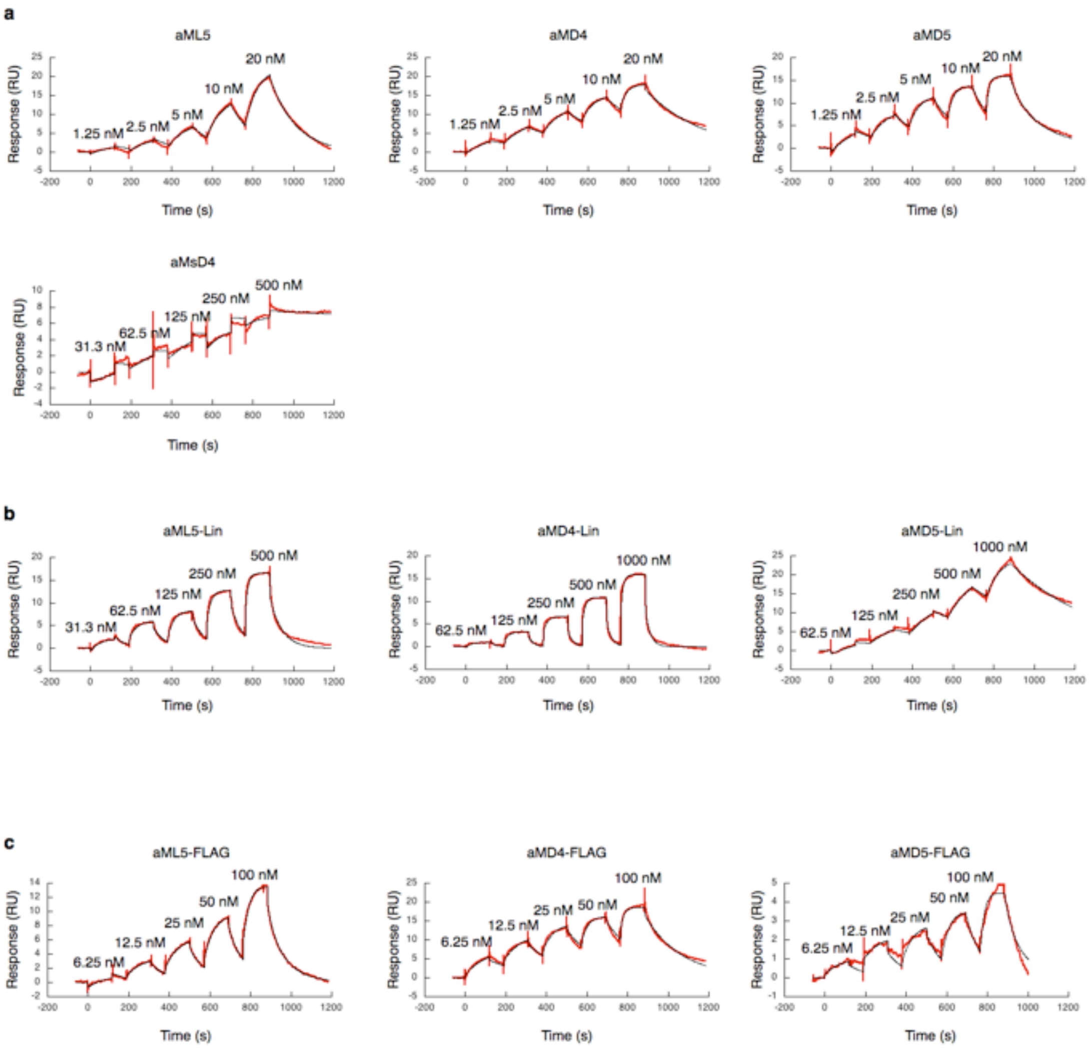

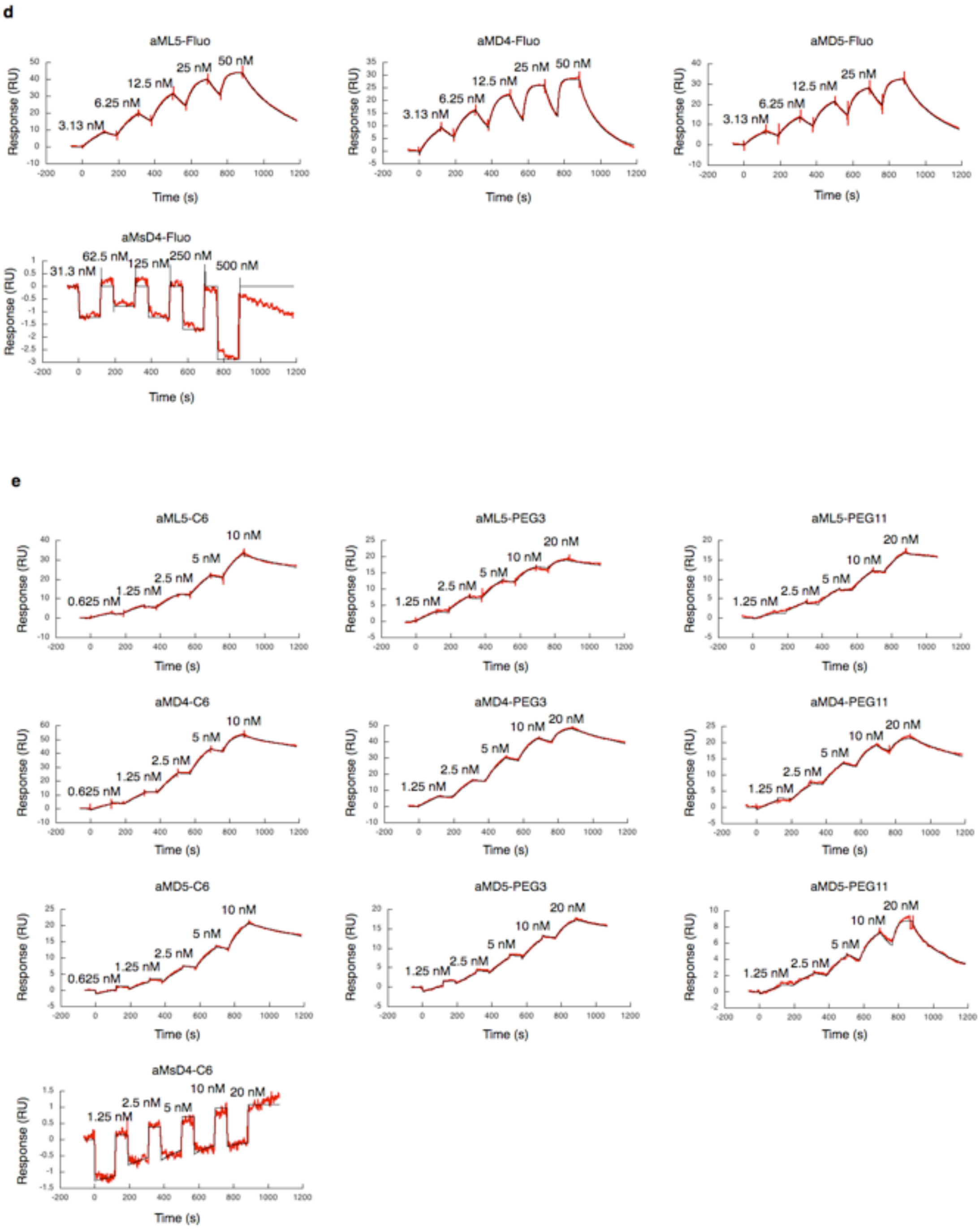

Supplementary Figure 3. SPR sensorgrams of (**a**) Met-binding macrocycles, (**b**) linear peptides, (**c**) FLAG-tagged macrocycles, (**d**) fluorescein-conjugated macrocycles and (**e**) dimeric macrocycles against human Met ectodomain-Fc chimera protein analyzed by Biacore T200. Measured and fitted curves are shown in red and black, respectively.

Supplementary Figure 4

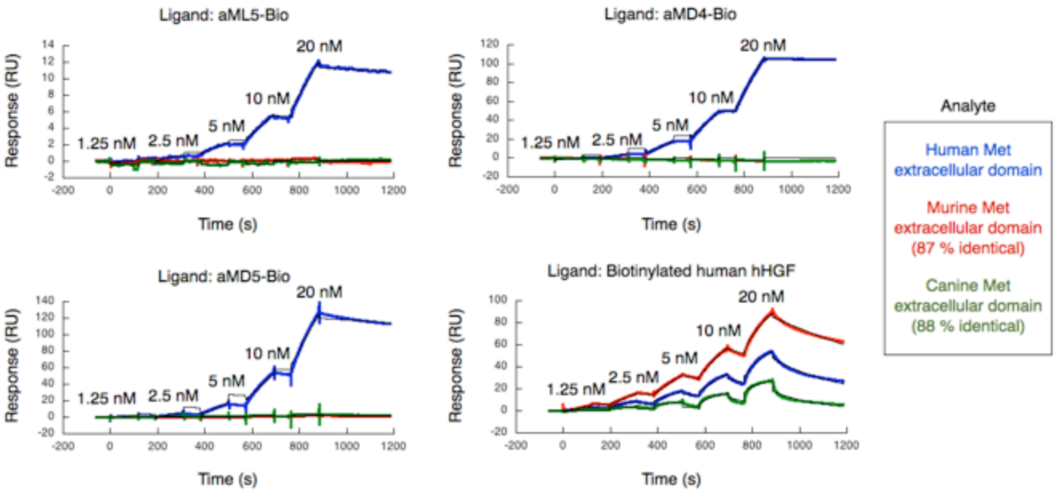

Supplementary Figure 4. SPR sensorgrams of human (blue), murine (red) and canine (green) Met ectodomains against C-terminal biotinylated macrocycles (aML5-Bio, aMD4-Bio and aMD5-Bio, whose sequences are shown in **Supplementary Table 2**) or biotinylated hHGF captured on a streptavidin-immobilized SPR sensor chip. Measured curves are shown in blue, red and green, and fitted curves are shown in black.

Supplementary Figure 5

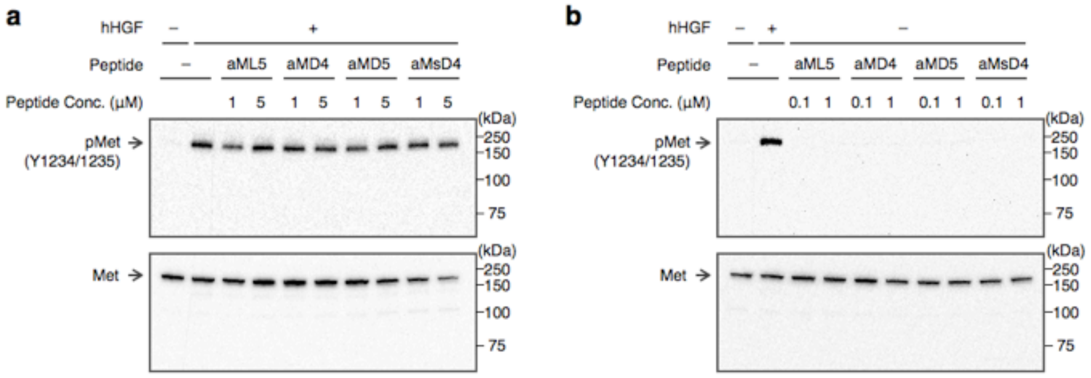

Supplementary Figure 5. Antagonistic and agonistic activities of monomeric macrocycles aML5, aMD4, aMD5 and aMsD4. **(a)** Western blotting of antagonistic ability of monomeric macrocycles. Human EHMES-1 cells were stimulated by hHGF (2 nM) or monomeric macrocycles (1 or 5 μM) for 15 mins, lysed and analyzed by western blotting using anti-pMet (Y1234/1235) mAb. (**b**) Western blotting of agonistic ability of Met-binding macrocycles. Human EHMES-1 cells were stimulated by hHGF (2 nM) or monomeric macrocycles (100 nM or 1 μM) for 15 mins, lysed and analyzed by western blotting using anti-pMet (Y1234/1235) mAb.

Supplementary Figure 6

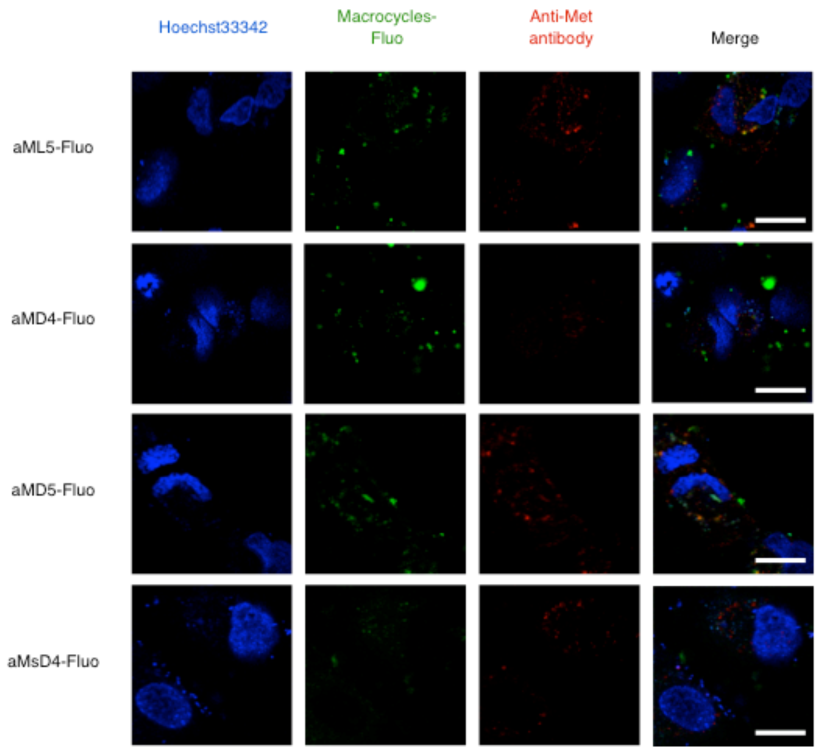

Supplementary Figure 6. Fluorescein-conjugated macrocycles on the cellular membranes of live Met-negative cells. EHMES-1 cells without overexpressing Met were treated with Hoechst33342, macrocycle-Fluo (aML5-Fluo, aMD4-Fluo, aMD5-Fluo, or aMsD4-Fluo) and an anti-Met rat mAb stained with an anti-rat mAb-Alexa-594 conjugate. Scale bars indicate 20 µm.

Supplementary Figure 7

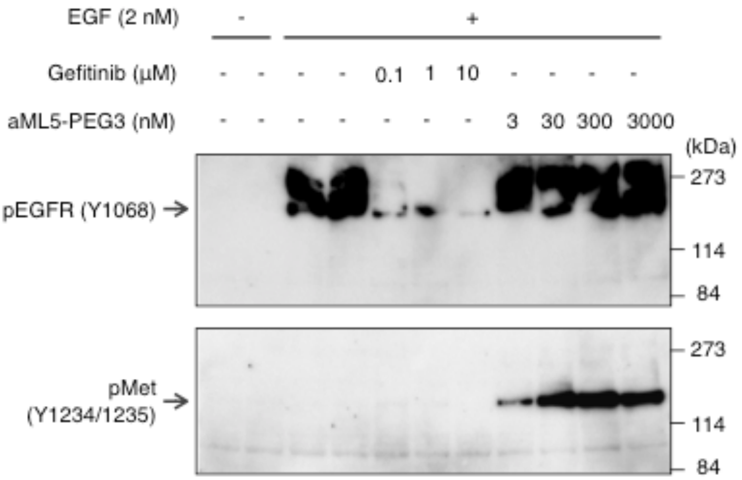

Supplementary Figure 7. Effect of dimer macrocycles on EGF-induced phosphorylation of EGFR. EHMES-1 cells were left unstimulated or stimulated with EGF in the presence or absence of the indicated concentrations of gefitinib (EGFR inhibitor) or aML5-PEG3. Cell lysates were subjected to SDS polyacrylamide gel electrophoresis and western blotting using anti-pEGFR (Y1068) antibody or anti-pMet (Y1234/1235) mAb.

Supplementary Figure 8

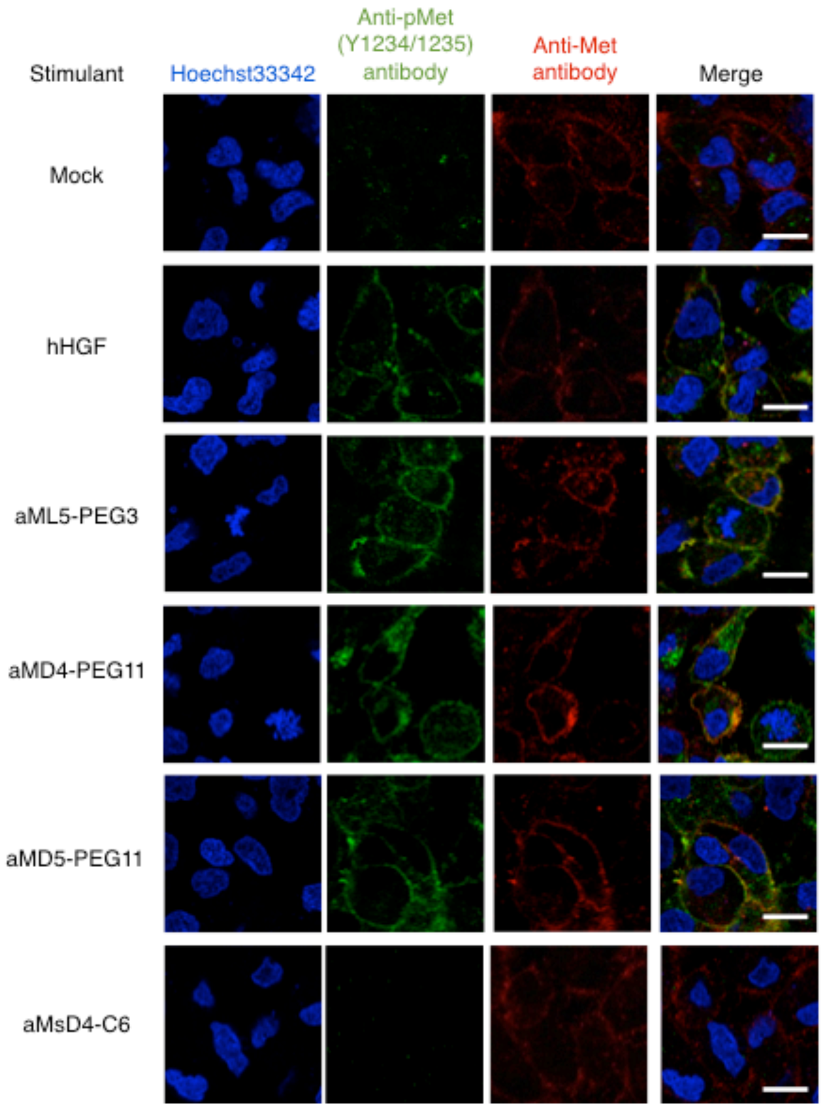

Supplementary Figure 8 Analysis of hHGF-induced Met dimerization by crosslinking on live cells. EHMES-1 cells were treated with hHGF for 60 min at 4 °C. After washing, cell surface proteins were crosslinked by BS3 for 60 min at 4 °C. Cells lysates were subjected to immunoprecipitation and western blotting using anti-Met antibody. Arrowhead and arrow indicate Met dimer and monomer corresponding to crosslinked αβ subunits, respectively. Asterisk indicates Met monomer corresponding to β subunit. Graph indicates relative level of Met dimer evaluated from the band intensity. Scale bars indicate 20 µm.

Supplementary Figure 9

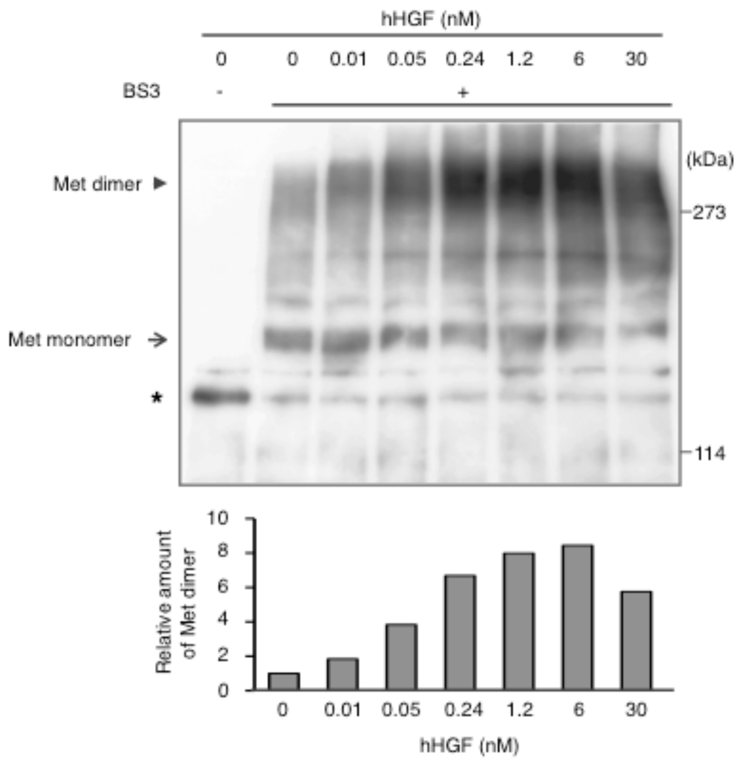

Supplementary Figure 9. Immunofluorescence microscopy images of phosphorylated Met on EHMES-1 cells. Cells were stimulated by 2 nM of hHGF or 100 nM of dimeric macrocycle and fixed by 4% PFA. Ectodomain and phosphorylated tyrosine kinase domain of Met were stained by pairs of anti-Met rat mAb/Alexa Fluor 594 anti-rat antibody (red) and anti-pY1234/1235 rabbit mAb/Alexa Fluor 488 anti-rabbit antibody (green), respectively. Cell nuclei were stained by Hoechst 33342 (blue). Scale bars: 20 μm.

Supplementary Figure 10

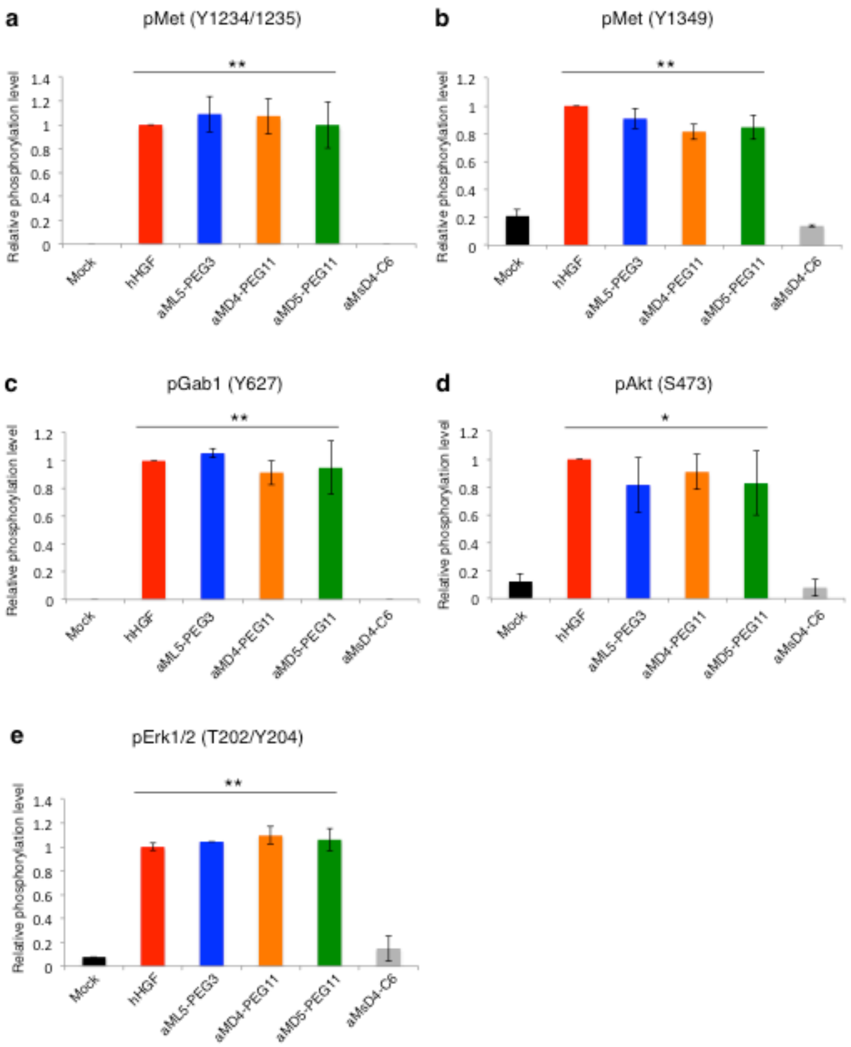

Supplementary Figure 10. Quantification of phosphorylation levels of the proteins in the Met signaling pathway. Starved EHMES-1 cells were treated with hHGF (2 nM) or the dimeric macrocycles (each supplied at 100 nM) for 10 min and phosphorylations of (**a**) Met (Y1234/1235), (**b**) Met (Y1349), (**c**) Gab-1 (Y627), (**d**) Akt (S473), and (**e**) Erk1/2 (T202/Y204) were analyzed by western blotting using their specific antibodies. Phosphorylation level of each protein was evaluated from the band intensity of the phosphorylated protein relative to that of the total protein. Standard deviation was calculated from the results of triplicate experiments. *p<0.05, **p<0.01 (Student's t-test) compared to Mock.

Supplementary Figure 11

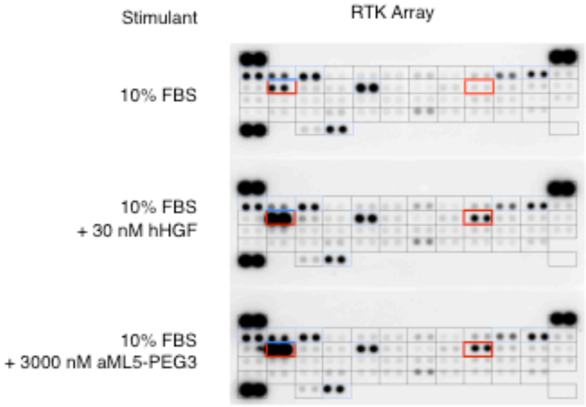

Pos Ctrl											Pos Ctrl
EGFR	ErbB2	ErbB3	ErbB4	FGFR1	FGFR2α	FGFR3	FGFR4	InsulinR	IGF-IR	Axl	Dtk
Mer	**Met**	MSPR	PDGFRα	PDGFRβ	SCFR	Flt-3	M-CSFR	**c-Ret**	ROR1	ROR2	Tie-1
Tie-2	TrkA	TrkB	TrkC	VEGFR1	VEGFR2	VEGFR3	MuSK	EphA1	EphA2	EphA3	EphA4
EphA6	EphA7	EphB1	EphB2	EphB4	EphB6	ALK	DDR1	DDR2	EphA5	EphA10	
Pos Ctrl		EphB3	RYK								Neg Ctrl

Supplementary Figure 11. Constitutively activated RTKs and the effect of excess amount of HGF or dimer macrocycles. EHMES-1 cells were cultured in the presence of 10% FBS (Mock) and constitutively activated RTKs were determined by phospho-RTK array. EGFR, ErbB3, Axl, PDGFRβ, RYK were constitutively serum-activated at a significant level. ErbB2 and IGF-IR were constitutively serum-activated at a weak level. Addition of excess amounts of HGF (30 nM) or aML5-PEG3 (3000 nM) did not inhibit these constitutively activated RTKs. Note that both HGF and aML5-PEG3 indirectly activated c-Ret through Met activation (also see Supplementary Fig. 12).

Supplementary Figure 12

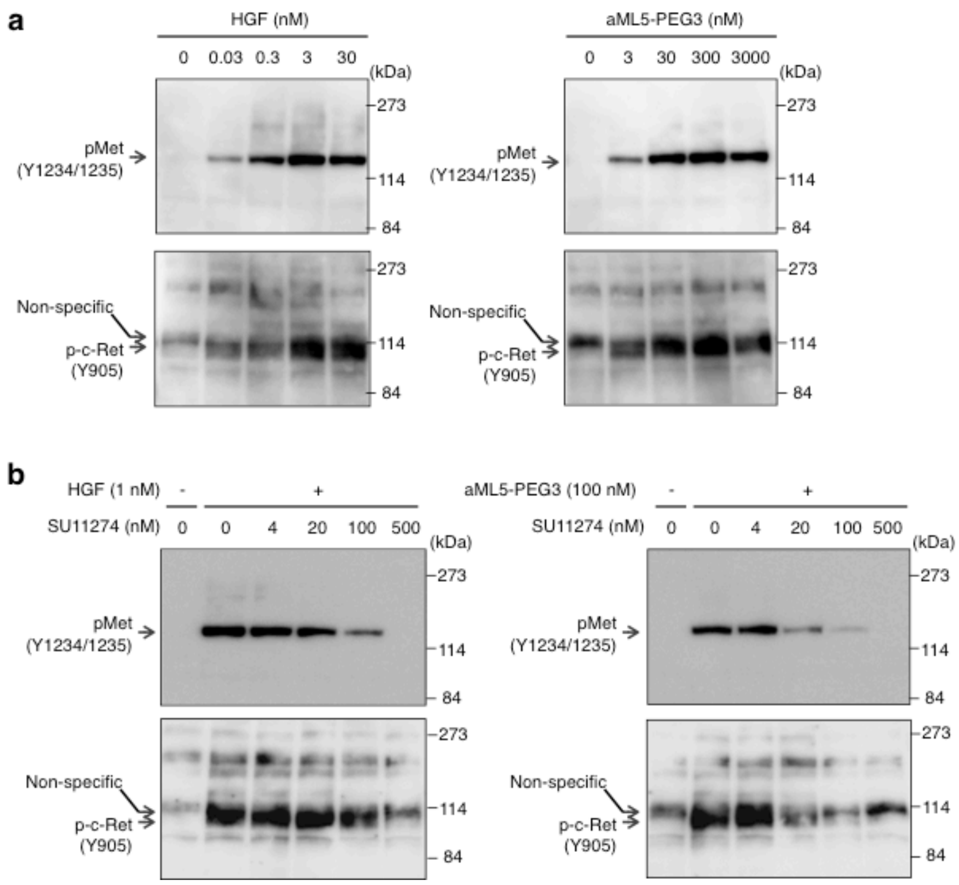

Supplementary Figure 12. Indirect activation of c-Ret via c-Met activation. (a) Correlation of Met phosphorylation and c-Ret phosphorylation induced by hHGF or aML5-PEG3. EHMES-1 cells were left unstimulated or stimulated with indicated concentrations of hHGF or aML5-PEG3. Cell lysates were subjected to SDS polyacrylamide gel electrophresis and western blotting using anti-pMet (Y1234/1235) mAb or anti-p-c-Ret (Y905) mAb. (b) Dependency of c-Ret phosphorylation on Met. EHMES-1 cells were left unstimulated or stimulated with 1 nM hHGF or 100 nM aML5-PEG3 in the presence or absence of indicated concentration of specific Met kinase inhibitor, SU11274. Cell lysates were subjected to SDS polyacrylamide gel electrophresis and western blotting as described above.

Supplementary Figure 13

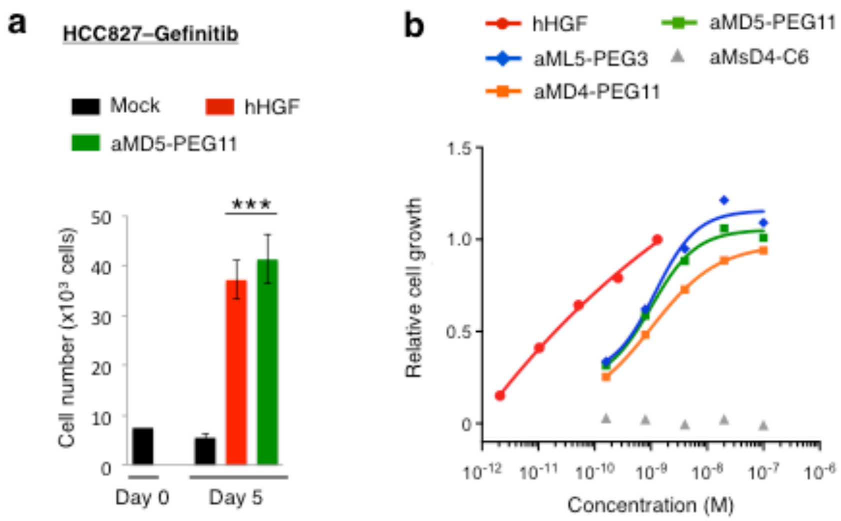

Supplementary Figure 13. Proliferation by dimeric macrocycles in gefitinib-treated human lung adenocarcinoma HCC827 cell line. (**a**) Cells were cultured in the presence of 1 μM gefitinib, with or without 1.3 nM hHGF (red) or 50 nM aMD5-PEG11 (green). Cell numbers were counted by means of an automated cell counter after 5 days culture (mean ± SD, n=4). $**p<0.01$, $***p<0.001$ (Student's t-test) compared to Mock. (**b**) Cells were cultured in the presence of 1 μM gefitinib, with or without (0.002, 0.01, 0.052, 0.26, 1.3 nM) or dimeric macrocycles (0.16, 0.8, 4, 20, 100 nM). After 4 days culture, cells were stained with crystal violet and relative cell growth was quantified by absorbance of 560 nm.

Supplementary Figure 14

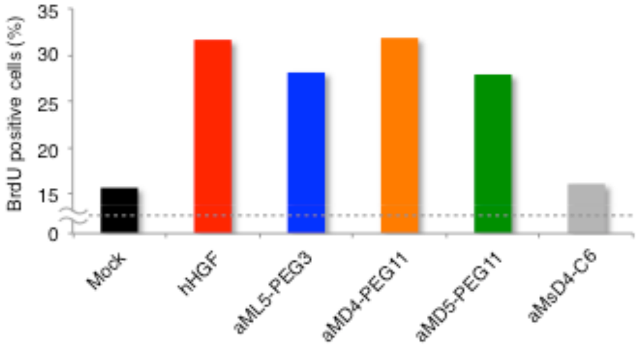

Supplementary Figure 14. Promotion of DNA synthesis in normal human pulmonary artery endothelial cells (HPAEC). Cells were cultured in the presence of 0.25 nM hHGF or 30 nM dimeric macrocycles. DNA synthesis was measured by BrdU incorporation into nuclei.

Supplementary Figure 15

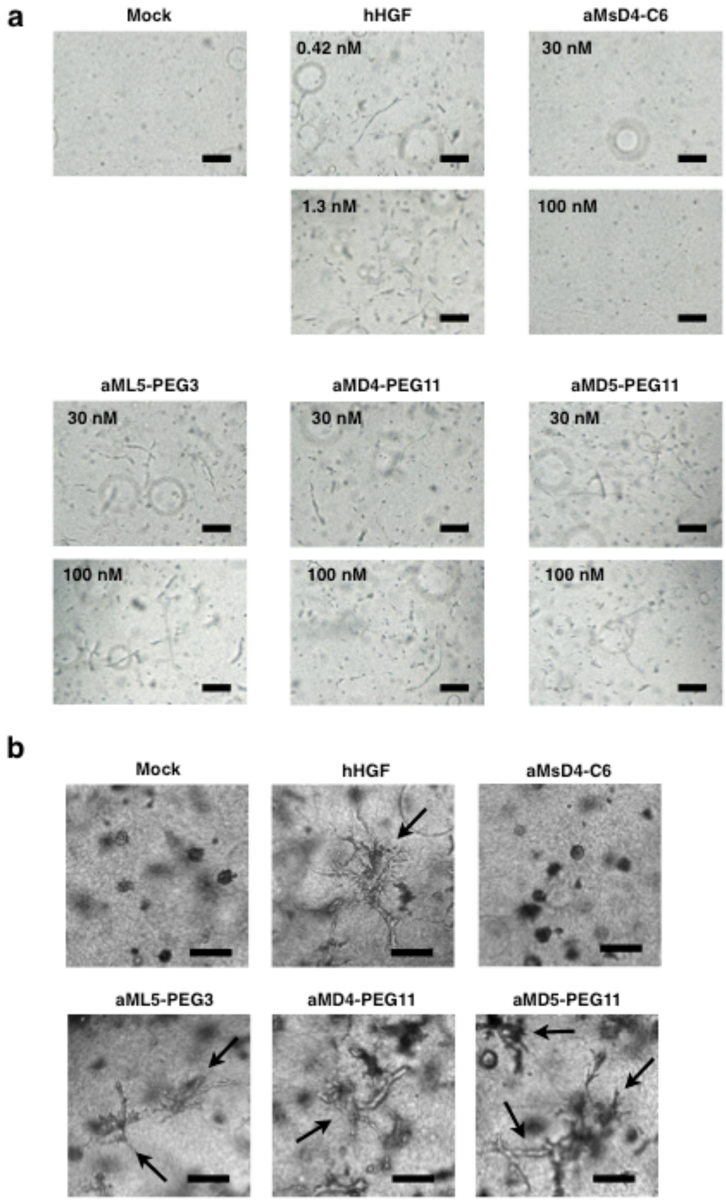

Supplementary Figure 15. Induction of tubular morphogenesis. (**a**) Wide ranging images of tubules formed by RPTEC. RPTEC encapsulated in collagen gels were cultured in the presence of 0.42 or 1.3 nM hHGF and 30 or 100 nM dimeric macrocycles over 8 days. Scale bars indicate 100 µm. (**b**) Tubules formed by HuCCT1 bile duct epithelial cells. HuCCT1 cells encapsulated in collagen gels were cultured in the presence of 0.38 nM hHGF or 20 nM dimeric macrocycles over 8 days. Typical tubules are indicated by arrows. Scale bars indicate 200 µm.

Supplementary Figure 16

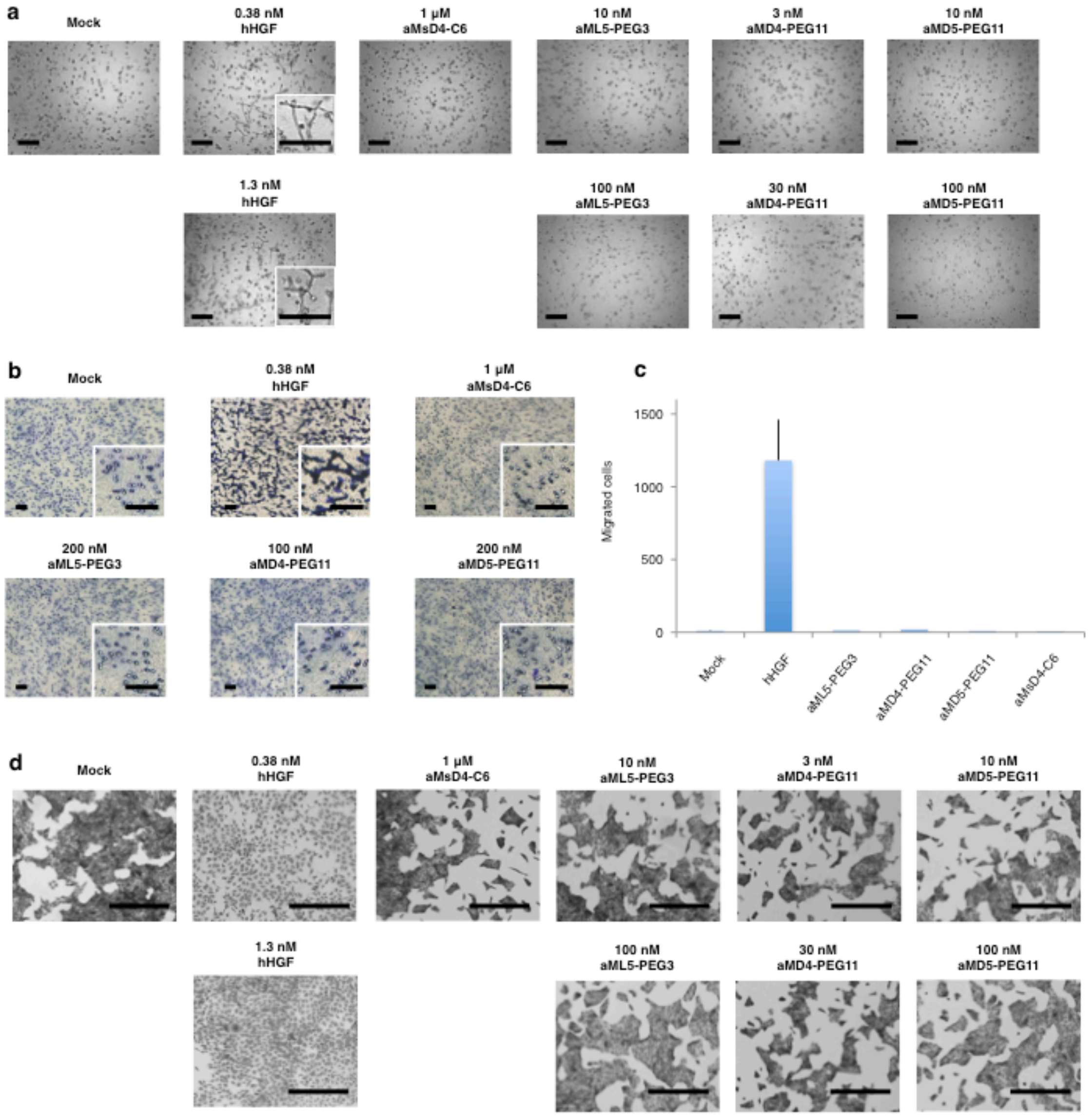

Supplementary Figure 16. Biological responses of murine and canine cells induced by hHGF or dimeric macrocycles. (**a**) Tubular morphogenesis assay using murine mammary epithelial TAC2 cells. Cells encapsulated in collagen gels were cultured for 7 days in medium supplemented with 1 μM hydrocortisone with or without hHGF or dimeric macrocycles. Scale bars indicate 500 μm. (**b, c**) Cellular migration of murine melanoma cells evaluated by a transwell assay. Cells were stimulated by hHGF or dimeric macrocycles over 16 h, and migrated cells were stained and quantified by crystal violet. (**b**) Representative images migrated murine melanoma cells. Scale bars

indicate 100 μm. (**c**) Number of migrated cells stimulated by hHGF or dimeric macrocycles. Standard deviation was calculated from the results of triplicate experiments. (**d**) Scatter assay using canine renal epithelial MDCK cells. Cells were incubated with hHGF or dimeric macrocycles and observed after 12 h culturing. Scale bars indicate 500 μm.

Supplementary Figure 17

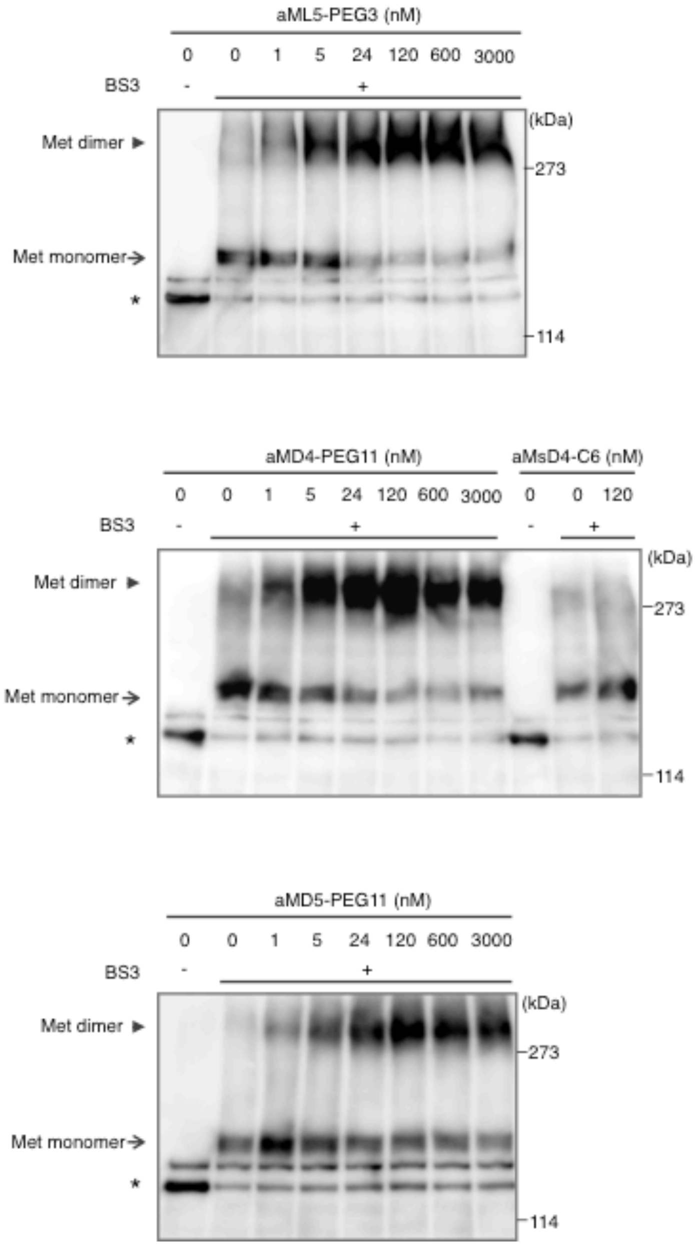

Supplementary Figure 17. Original blot images for Figure 2e.

Supplementary Figure 18

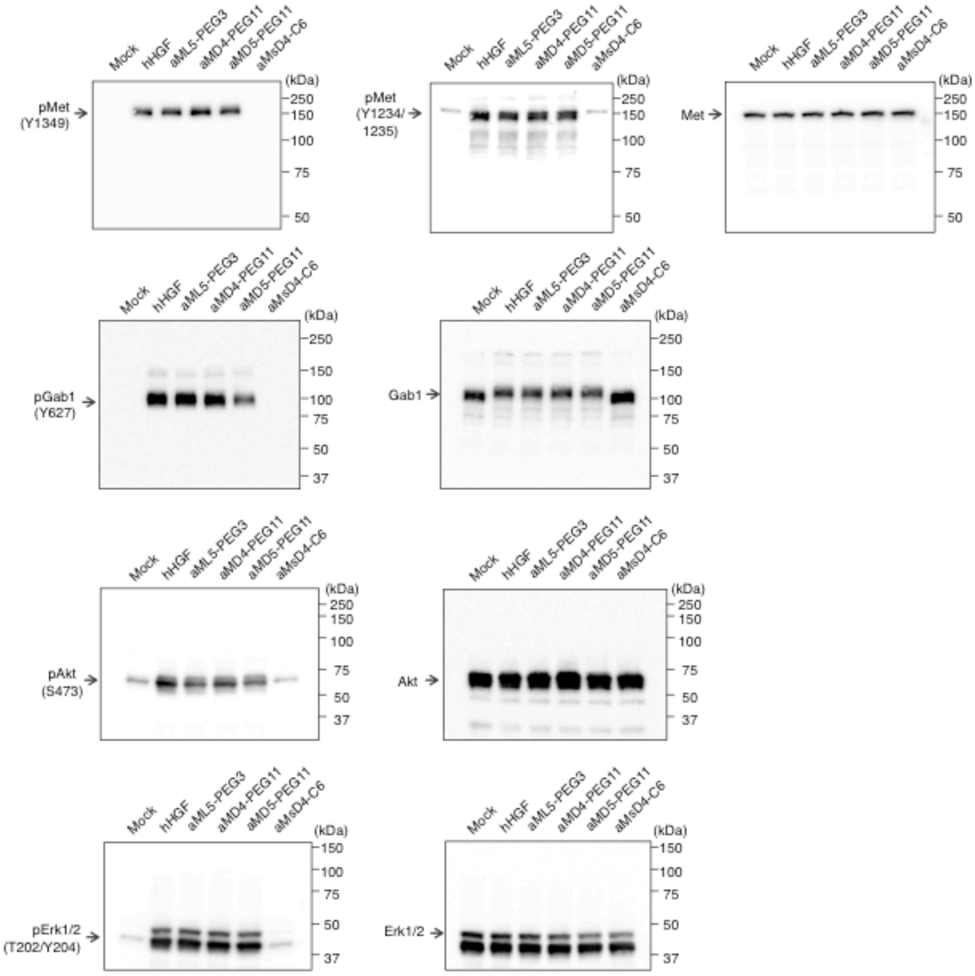

Supplementary Figure 18. Original blot images for Figure 2f.

Supplementary Table 1. Sequences of thioether macrocycles targeting human Met ectodomain, selected from L- (a) and D-libraries (b) by the RaPID system.

a L-library

S

Ac-^{L}Y(Random region)C-GSGSGS

Macrocycle	Random region	Frequency
**aML5**	ISWNEFNSPNWRFIT	3/23
	WYYHFKGYYTPIK	2/23
	WYYKYNQTWHLIQ	2/23
	VVPNPYFTVIA	2/23
	YYIAPHPYFTVIQYT	1/23
	IAPKPYFVVVA	1/23
	IVEPPYFIVVA	1/23
	LAWESFDSKDWHPV	1/23
	LTWSTYDSRHWVFKT	1/23
	ITFNYKTHEVSSYK	1/23
	LQYNDKTHEVWQYA	1/23
	LTFYGPPTFEVYNFD	1/23
	RFIQPKTHFIPEWF	1/23
	FYWYRLTQSYRKLA	1/23
	IYYWIRNDYVDY	1/23
	WINRSTLEIFLYTW	1/23
	VRHTVLSWFKDYTH	1/23
	WYYRFDFRWTPIH	1/23

b D-library

S

Ac-^{D}Y(Random region)C-GSGSGS

Macrocycle	Random region	Frequency
**aMD5**	WYYAWDQTYKAFP	5/23
**aMD4**	RQFNRRTHEVWNLD	2/23
	VFYYDGRQRWTPLPN	2/23
	WYFNFRSQWIPAP	1/23
	WYYSYANLWKPIQ	1/23
	QWYFNFKNTWTLI	1/23
	YYYTFTQQWRPTP	1/23
	YYLDYQPTRRYLA	1/23
	WYYSFDQRWVPSY	1/23
	LYYSFDQKWKNVP	1/23
	PTFYYNYRSEWVLTS	1/23
	TVHEGWPTFIVWHLD	1/23
	LWVWNVISL	1/23
	FLVSGYPTFSVIHWN	1/23
	WVYIISYLLYGP	1/23
	LKWNDFNSKQWKFSF	1/23
	GDTHQSPPTR	1/23

Supplementary Table 2. Sequences and kinetic constants of the macrocycles, C-terminal labeled macrocycles (a), and dimeric macrocycles (b) used in the study.

a

Macrocycle	Sequence	k_a (10^6 $M^{-1}s^{-1}$)	k_d (10^{-2} s^{-1})	K_D (nM)
aML5	S<br>Ac-^{L}YISWNEFNSPNWRFITCG-NH_2	0.43	0.81	19
aMD4	S<br>Ac-^{D}YRQFNRRTHEVWNLDCG-NH_2	1.4	0.34	2.4
aMD5	S<br>Ac-^{D}YWYYAWDQTYKAFPCG-NH_2	4.8	1.1	2.3
aMsD4	S<br>Ac-^{D}YERVNHLFRNQTWDRCG-NH_2		No binding	
aML5-Lin	Ac-^{L}YISWNEFNSPNWRFITAG-NH_2	0.013	1.7	1300
aMD4-Lin	Ac-^{D}YRQFNRRTHEVWNLDAG-NH_2	0.55	4.0	750
aMD5-Lin	Ac-^{D}YWYYAWDQTYKAFPAG-NH_2	0.012	0.23	190
aML5-FLAG	S<br>Ac-^{L}YISWNEFNSPNWRFITC^{β}A^{β}ADYKDDDDK-NH_2	0.44	2.6	58
aMD4-FLAG	S<br>Ac-^{D}YRQFNRRTHEVWNLDC^{β}A^{β}ADYKDDDDK-NH_2	0.59	0.58	9.7
aMD5-FLAG	S<br>Ac-^{D}YWYYAWDQTYKAFPC^{β}A^{β}ADYKDDDDK-NH_2	1.6	2.3	14
aML5-Fluo	S NHCO-Fluo<br>Ac-^{L}YISWNEFNSPNWRFITCG^{β}AK-NH_2	1.1	0.46	4.2
aMD4-Fluo	S NHCO-Fluo<br>Ac-^{D}YRQFNRRTHEVWNLDCG^{β}AK-NH_2	0.78	0.50	6.4
aMD5-Fluo	S NHCO-Fluo<br>Ac-^{D}YWYYAWDQTYKAFPCG^{β}AK-NH_2	2.4	1.1	4.6
aMsD4-Fluo	S NHCO-Fluo<br>Ac-^{D}YERVNHLFRNQTWDRCG^{β}AK-NH_2		No binding	
aML5-Bio	S NHCO-PEO4-Bio<br>Ac-^{L}YISWNEFNSPNWRFITCG^{β}AK-NH_2	N.D.	N.D.	N.D.
aMD4-Bio	S NHCO-Bio<br>Ac-^{D}YRQFNRRTHEVWNLDCG^{β}AK-NH_2	N.D.	N.D.	N.D.
aMD5-Bio	S NHCO-Bio<br>Ac-^{D}YWYYAWDQTYKAFPCG^{β}AK-NH_2	N.D.	N.D.	N.D.

b

Macrocycle	Sequence	Linker	k_a (10^6 $M^{-1}s^{-1}$)	k_d (10^{-2} s^{-1})	K_D (nM)
aML5-C6	Ac-^{L}YISWNEFNSPNWRFITCG$^{\beta}$AC-NH$_2$	BMH	0.079	0.073	9.2
aML5-PEG3	S–Linker–S	BMPEG3	0.22	0.042	1.9
aML5-PEG11	Ac-^{L}YISWNEFNSPNWRFITCG$^{\beta}$AC-NH$_2$	BMPEG11	0.098	0.031	3.1
aMD4-C6	Ac-^{D}YRQFNRRTHEVWNLDCG$^{\beta}$AC-NH$_2$	BMH	2.4	0.057	0.24
aMD4-PEG3	S–Linker–S	BMPEG3	1.1	0.070	0.64
aMD4-PEG11	Ac-^{D}YRQFNRRTHEVWNLDCG$^{\beta}$AC-NH$_2$	BMPEG11	1.2	0.11	0.90
aMD5-C6	Ac-^{D}YWYYAWDQTYKAFPCG$^{\beta}$AC-NH$_2$	BMH	0.064	0.068	11
aMD5-PEG3	S–Linker–S	BMPEG3	0.12	0.054	4.7
aMD5-PEG11	Ac-^{D}YWYYAWDQTYKAFPCG$^{\beta}$AC-NH$_2$	BMPEG11	5.3	1.1	2.1
aMsD4-C6	Ac-^{D}YERVNHLFRNQTWDRCG$^{\beta}$AC-NH$_2$ – S–Linker–S – Ac-^{D}YERVNHLFRNQTWDRCG$^{\beta}$AC-NH$_2$	BMH	No binding		

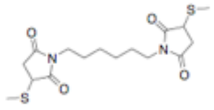
BMH

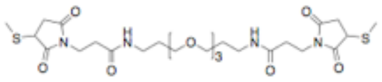
BMPEG3

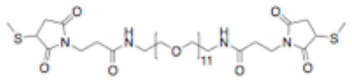
BMPEG11

Supplementary Table 3. Kinetic constants of the biotinylated monomeric macrocycles and hHGF targeting human, murine, and canine Met ectodomains.

Ligand	Analyte	k_a (10^5 $M^{-1}s^{-1}$)	k_d (10^{-3} s^{-1})	K_D (nM)
aML5-Bio	Human Met	0.043	0.15	35
	Murine Met		No binding	
	Canine Met		No binding	
aMD4-Bio	Human Met	12	0.20	0.18
	Murine Met		No binding	
	Canine Met		No binding	
aMD5-Bio	Human Met	1.8	3.1	17
	Murine Met		No binding	
	Canine Met		No binding	
Biotinylated hHGF	Human Met	11	5.3	4.9
	Murine Met	3.5	1.2	3.4
	Canine Met	19	5.8	3.1

Supplementary Table 4. Proteins immunoprecipitated by FLAG-tagged macrocycles.

FLAG-macrocycle (Bait)	Protein (Prey)	Species	Localization	Sequences of identified fragments	Start-stop	Frequency
aML5-FLAG	Met	Hepatocyte growth factor receptor	Cellular membrane	AFFMLDGILSK	825-835	4/4
aMD4-FLAG	Met	Hepatocyte growth factor receptor	Cellular membrane	SEMNVNMK	33-40	2/4
				DGFMFLTDQSYIDVLPEFRDSYPIK	224-248	4/4
				YVNDFFNK	379-386	1/4
				SFISGGSTITGVGK	752-765	4/4
				AFFMLDGILSK	825-835	4/4
				GNDIDPEAVK	852-860	3/4
				GNDIDPEAVKGEVLK	852-866	4/4
				LNSELNIEWK	903-912	4/4
				QAISSTVLGK	913-922	4/4
				DLIGFGLQVAK	1180-1190	2/4
				VADFGLARDMYDK	1220-1232	3/4
				WMALESLQTQK	1249-1259	1/4
	PDIA3	Protein disulfide-isomerase A3	ER membrane	DASIVGFFDDSFSEAHSEFLK	153-173	3/4
				SEPIPESNDGPVK	367-379	1/4
aMD5-FLAG	NDUFA5	NADH dehydrogenase [ubiquinone] 1 alpha subcomplex subunit 5	Mitochondrial membrane	YTEQITNEK	47-85	4/4

Supplementary Table 5. Summary of biological responses of human, mouse, and canine cells stimulated by dimeric macrocycles or hHGF.

Stimulant	Human cells					Mouse cells		Canine cells
	Met phosphorylation	Cell growth	Migration	Wound healing	Tubular morphogenesis	Migration	Tubular morphogenesis	Scatter
hHGF	○	○	○	○	○	○	○	○
aML5-PEG3	○	○	○	○	○	×	×	×
aMD4-PEG11	○	○	○	○	○	×	×	×
aMD5-PEG11	○	○	○	○	○	×	×	×
aMsD4-C6	×	×	×	×	×	×	×	×
